# Hyperelastic, shape‐memorable, and ultra‐cell‐adhesive degradable polycaprolactone‐polyurethane copolymer for tissue regeneration

**DOI:** 10.1002/btm2.10332

**Published:** 2022-05-05

**Authors:** Suk‐Min Hong, Ji‐Young Yoon, Jae‐Ryung Cha, Junyong Ahn, Nandin Mandakhbayar, Jeong Hui Park, Junseop Im, Gangshi Jin, Moon‐Young Kim, Jonathan C. Knowles, Hae‐Hyoung Lee, Jung‐Hwan Lee, Hae‐Won Kim

**Affiliations:** ^1^ Institute of Tissue Regeneration Engineering (ITREN) Dankook University Cheonan Chungcheognam‐do Republic of Korea; ^2^ Department of Nanobiomedical Science and BK21 PLUS NBM Global Research Center for Regenerative Medicine Dankook University Cheonan Chungcheognam‐do Republic of Korea; ^3^ Department of Chemistry, College of Science and Technology Dankook University Cheonan Chungcheognam‐do Republic of Korea; ^4^ UCL Eastman‐Korea Dental Medicine Innovation Centre Dankook University Cheonan Chungcheognam‐do Republic of Korea; ^5^ Cell & Matter Corporation Cheonan Chungcheongnam‐do Republic of Korea; ^6^ Department of Biomaterials Science College of Dentistry, Dankook University Cheonan Chungcheognam‐do Republic of Korea; ^7^ Samyang Corporation Yuseong‐gu Daejeon Republic of Korea; ^8^ Department of Oral and Maxillofacial Surgery College of Dentistry, Dankook University Cheonan Chungcheongnam‐do Republic of Korea; ^9^ Division of Biomaterials and Tissue Engineering Eastman Dental Institute, Royal Free Hospital London UK; ^10^ The Discoveries Centre for Regenerative and Precision Medicine Eastman Dental Institute, University College London London UK; ^11^ Cell & Matter Institute Dankook University Cheonan Chungcheongnam‐do Republic of Korea

**Keywords:** cell adhesion, hyperelasticity, polyurethane, shape memory, tissue regeneration

## Abstract

Novel polycaprolactone‐based polyurethane (PCL‐PU) copolymers with hyperelasticity, shape‐memory, and ultra‐cell‐adhesion properties are reported as clinically applicable tissue‐regenerative biomaterials. New isosorbide derivatives (propoxylated or ethoxylated ones) were developed to improve mechanical properties by enhanced reactivity in copolymer synthesis compared to the original isosorbide. Optimized PCL‐PU with propoxylated isosorbide exhibited notable mechanical performance (50 MPa tensile strength and 1150% elongation with hyperelasticity under cyclic load). The shape‐memory effect was also revealed in different forms (film, thread, and 3D scaffold) with 40%–80% recovery in tension or compression mode after plastic deformation. The ultra‐cell‐adhesive property was proven in various cell types which were reasoned to involve the heat shock protein‐mediated integrin (α5 and αV) activation, as analyzed by RNA sequencing and inhibition tests. After the tissue regenerative potential (muscle and bone) was confirmed by the myogenic and osteogenic responses in vitro*,* biodegradability, compatible in vivo tissue response, and healing capacity were investigated with in vivo shape‐memorable behavior. The currently exploited PCL‐PU, with its multifunctional (hyperelastic, shape‐memorable, ultra‐cell‐adhesive, and degradable) nature and biocompatibility, is considered a potential tissue‐regenerative biomaterial, especially for minimally invasive surgery that requires small incisions to approach large defects with excellent regeneration capacity.

## 1 INTRODUCTION

The growing demand for versatile, biocompatible synthetic polymers in biomedical engineering and medical devices has galvanized research beyond the biocompatibility and tissue‐regenerative ability of FDA‐approved synthetic polymers such as polycaprolactone (PCL), poly(methyl methacrylate) (PMMA), poly(lactic‐co‐glycolic acid) (PLGA), and polyurethanes (PU) and toward the design of more tailorable polymeric materials with enhanced biological and mechanical properties.^1^, ^2^, ^3^, ^4^, ^5^ PU‐based polymers have recently attracted substantial interest for use in various tissue‐regenerative processes, including muscle, cartilage, blood vessel and bone regeneration, because of their tunable properties, such as biodegradability, elasticity and resistance to flex fatigue.^6^, ^7^, ^8^, ^9^, ^10^ However, poor cell adhesiveness and the need for further bio‐friendly surface modification due to the innate absence of functional groups from their original structures remain obstacles to the use of PU‐based polymers as substrates for clinical tissue regeneration along with low biodegradability,^11^, ^12^, ^13^, ^14^, ^15^ while cell attachment is considered the essential first step to trigger proliferation, migration and differentiation, which are prerequisites for tissue regeneration.^16^, ^17^

Myriad strategies have been attempted to increase the cell adhesiveness of PU‐based polymers for use in clinical settings.^18^, ^19^, ^20^ The aromatic diisocyanates, which has an isocyanate (N=C=O) group directly attached to the aromatic ring, could improve the cell‐adhesive functionalities when used in synthesizing PU polymers and enhance mechanical properties,^21^, ^22^, ^23^, ^24^, ^25^, ^26^ but the utilization of these materials in clinical settings is limited due to the low degradation rate and to tumorigenesis induced by degradation or unreacted monomers.^27^, ^28^ Instead, aliphatic diisocyanates has been introduced as a component of PU polymers due to its superior biocompatibility without toxic degradation byproducts, but they had compromised mechanical properties with moderate cell adhesiveness.^29^, ^30^ Although there have been a lot of efforts to enhance biological and mechanical properties of aliphatic isocyanate‐based PU polymers by structural or component modifications, they are still suboptimal.^31^, ^32^

Shape‐memory biomaterials have also received attention as morphologically responsive materials with a variety of potential applications, particularly in biomedical devices for minimally invasive surgery and the delivery of therapeutics and cells for tissue engineering.^33^, ^34^, ^35^, ^36^, ^37^ Shape‐memory features have been associated mainly with metals and detected with synthetic polymer materials and some biological substrates.^38^, ^39^, ^40^, ^41^ To combine the merits of both elasticity and shape‐memory properties, shape‐memorable PUs has been developed, but the mechanical strength, shape‐memory behavior, and cell‐adhesive properties are unsatisfactory for clinical utilization.^37^, ^42^ To solve the above issues, PCL diol‐1,6‐hexamethylene diisocyanate (HDI)‐based PU polymers, as an aliphatic isocyanate‐based PUs, were developed for biocompatible shape‐memory PUs and isosorbide was added as reinforcement for enhanced mechanical properties, but the cell‐adhesive property was still subprime.^22^, ^25^, ^43^ In particular, even though PCL and isosorbide‐based PUs with shape‐memorable, degradable, and elastic properties have been reported, those were still lack of mechanical and biological evaluations from in vitro and in vivo studies for being utilized in tissue regeneration, leading to the investigation of new PCL‐PU copolymer for biomedical application.

The isosorbide is known as chiral and rigid molecules, and the innocuous nature ensures its use as an biocompatible alternative to petroleum‐based polymer, opening up the possibility of utilization for medical devices.^44^ But bare isosorbide had the low reactivity of the secondary hydroxyl groups, limiting participation in the copolymer synthesis.^45^, ^46^ To tackle above issues, various isosorbide derivatives with enhanced reactivity have been developed, but those were still suboptimal in the aspect of mechanical properties as well as cellular‐functionality for tissue regeneration.^47^, ^48^ Here, two newly synthesized isosorbide derivatives (propoxylated or ethoxylated isosorbide) with the enhanced reactivity were introduced for fabricating polyurethanes polymer over bare isosorbide. By increasing the reactivity and participation in the polymer structure, they had enhanced elastic properties as well as a stable shape transition temperature (*T*_m_) around body temperature. Interestingly, specific isosorbide‐derivative (propoxylated isosorbide)‐incorporated PCL‐PU (renamed as ISB‐P) was shown to have significantly enhanced cell‐adhesive properties for various cell types, including mesenchymal stem cell, compared with that of other shape‐memory PUs and even that of FDA‐approved PCL. A novel stem cell‐adhesive mechanism was elucidated with RNA sequence analysis, siRNA study and functional inhibitor study, revealing adhesion by heat shock protein‐mediated integrin α5 and αV. Comparison of the in vitro tissue (muscle and bone) regenerative potential with that of PCL after matching initial cell‐adhesive properties presented similar muscle maturation and bone differentiation. In vivo studies revealed biocompatibility, absence of major organ toxicity, tissue(bone)‐regenerative efficacy as well as shape‐memory properties in sinus and femur of live rabbit. Thus, the current isosorbide‐derivative PCL‐PU (ISB‐P) is considered a promising platform for tissue regeneration, especially for tissue defects with small openings connected to large defects, and is envisaged to extend to other biomedical applications that require repair and regeneration with volumetric self‐adjustment.

## 2 RESULTS AND DISCUSSION

### 2.1 Synthesis of versatile, hyperelastic, shape‐memorable PCL‐PU biomaterials

PCL diol‐HDI‐based PUs were synthesized by one‐shot catalyst‐free bulk polymerization and named ISB‐2′ (from original isosorbide), ISB‐P (from propoxylated isosorbide) and ISB‐E (from ethoxylated isosorbide), depending on their isosorbide derivatives (Table S1 and Figure S1). Isosorbide‐free PCL‐PU was also fabricated as synthesis control, consisting of PCL and HDI with the ratio of 1:1 without isosorbide. PCL diol and HDI were selected to achieve various basic tissue‐regenerative characteristics, such as biocompatibility, biodegradability (from PCL), and elasticity (from PU functional groups induced by HDI and alcohol of PCL or isosorbide derivative), after step‐growth polymerization. Aromatic diisocyanates was originally used to synthesize PUs, but their degradation products (e.g., 2,4‐diaminotoluene and 4,4′‐methylenedianiline) and unreacted isocyanates (e.g., toluene diisocyanate or methylene diphenyl diisocyanate) were potentially tumorigenic, limiting their application as implanted biomaterials.^49^, ^50^, ^51^, ^52^ To date, aliphatic diisocyanates, having less toxic or tumorigenic potential than aromatic diisocyanates, was synthesized into PU from PCL diol‐HDI but induced a decrease in mechanical stability. Therefore, isosorbide or its derivative was added to compensate for the reduced mechanical properties of PU.^21^, ^22^, ^23^, ^24^, ^25^, ^26^ Here, newly synthesized isosorbide derivatives (ethoxylated isosorbide and propoxylated isosorbide) were introduced as additive components and compared with bare isosorbide to test whether PU synthesized based on each isosorbide with PCL diol‐HDI had hyperelasticity, stable shape‐memory properties near body temperature as well as enhanced biological properties. By increasing the reactivity and participation of newly synthesized isosorbide derivatives in polymer structures, a bicyclic isosorbide structure was designed to increase the mechanical properties and decrease the shape transition temperature (*T*_m_) near 37.5°C ± 0.5 by reducing the free volume in the polymer structure.

^1^H NMR and FTIR results confirmed the as‐designed chemical structures (Figure S2). In ^1^H NMR spectra, the peak at 3.15 ppm corresponds to the amine proton (purple) of the urethane N—H moiety, and the bicyclic methylene proton (blue) at 5.18–4.21 ppm. The alkyl chain (orange) peak at 3.63–3.41 ppm. The propylene group (red) of ISB‐P peak at 1.13 and 1.24. The alkylene protons (green) of PCL diol appeared at 4.09, 3.89, 2.34, 1.62, 1.38, and 0.99 ppm, and peaks corresponding to the methylene protons (yellow) of HDI appeared at 1.48 and 1.29 ppm. FTIR results showed urethane bonds [(‐NH‐COO‐) at 3435–3282 (N‐H stretching), 1724 (C=O stretching), and 1527 (N‐H bending and C‐N stretching) cm^−1^ from FTIR] without unreacted isocyanate in HDI (2270 cm^−1^) in the PCL‐PU polymers (Figure S2), and gel permeation chromatography revealed an increase in the average molecular weight (*M*_w_) from the new isosorbide derivatives [ISB‐P (139,744) and ISB‐E (123,786)] compared to the original isosorbide [ISB‐2′ (103,336)] and isosorbide‐free [ISB‐free (89,534)]^45^ due to the higher primary alcohol reactivity of isosorbide derivatives for polymerization (Table S2). In addition, this difference in molecular weight among groups has the potential to affect the physicochemical properties, especially the mechanical performances.^53^, ^54^, ^55^ Polydispersity index was ~2 in ISB‐2′ because secondary alcohol in isosorbide is less reactive for PCL‐PU polymerization than primary alcohol and 2‐propanol in ISB‐E and ISB‐P, respectively.^56^

**FIGURE 1:**
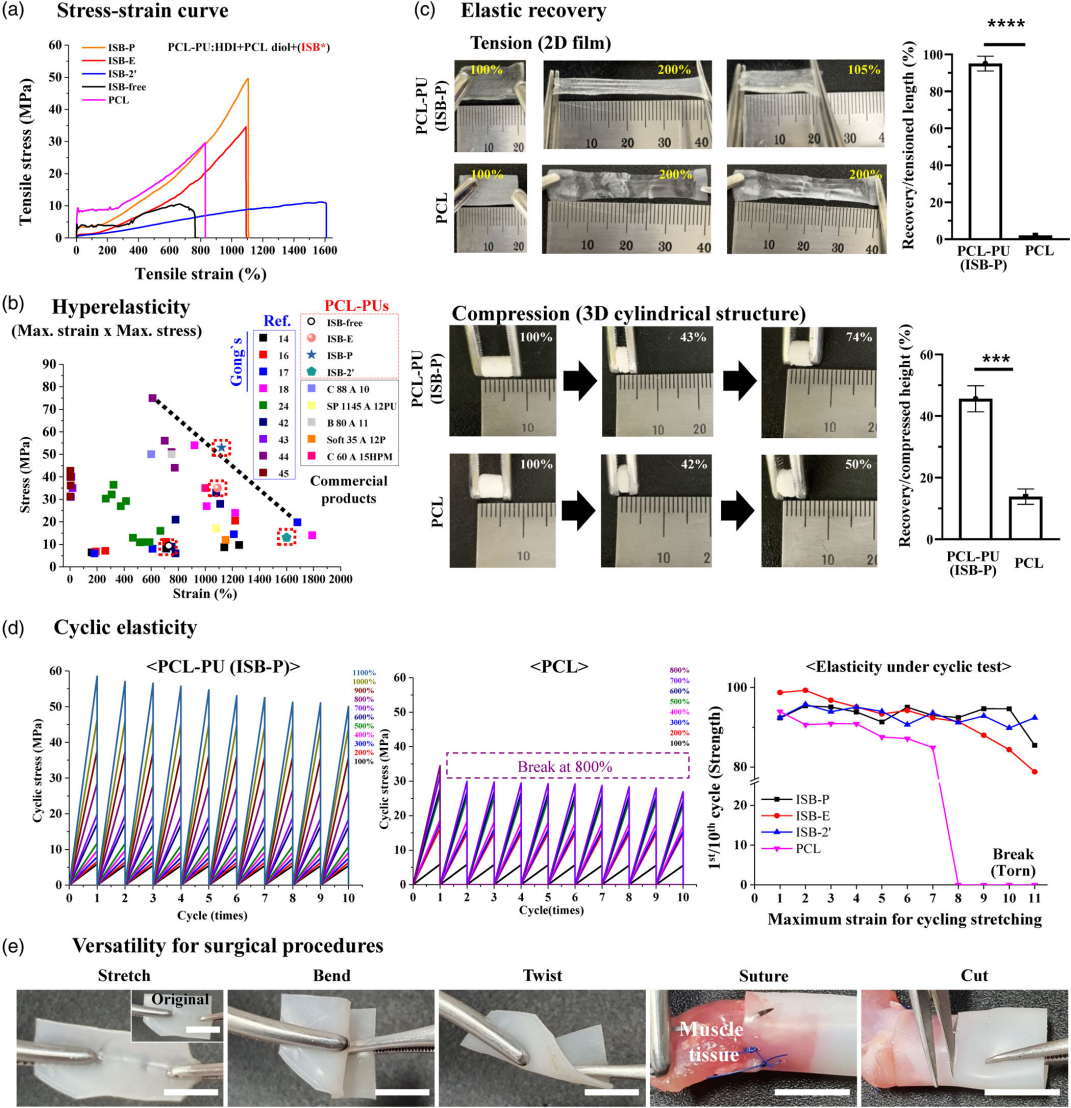
Hyperelastic and surgically compatible PCL‐PU biomaterials. (a) Stress–strain curve in tension from PCL‐PU and PCL. Depending on the isosorbide derivatives in synthesizing PCL‐PU, subtype of PCL‐PU was named as ISB‐P, ISB‐E, ISB‐2′ and ISB‐free. (b) Various elasticity plots of references 21, 23, 24, 25, 32, 57, 58, 59, 60, commercially available PU and PCL‐PU synthesized, displaying hyperelastic properties, as approximately determined by (maximum stress) × (strain at break) values, of ISB‐P with 50 MPa maximum stress and ~1100% of strain at break. Black dot line indicated controllable characteristics of ISB‐P by the ratio of each component of ISB‐P. Black dot rectangle (experimental value tested), black line rectangle (the value of commercial product given by company), others (according to the ref. including ours [Gong's]). ISB‐free was excluded for further experiment due to poor elasticity. (c) Bulk elastic recovery test using tension and compression was performed on film and 3D scaffold, respectively, revealing enhanced elasticity in PCL‐PU (ISB‐P) than PCL (*n* = 3, ***p < 0.001, ****p < 0.0001 by *t* test). (d) Dynamic mechanical behavior under cyclic tensile loading conditions (10 cycles, 100%–1100% strain, 10 mm/min), displaying maintenance of hyperelasticity of ISB‐P (80%–95% of initial strength) during 10 times cyclic tension against up to maximum tensile strain (~1100%). In contrast, PCL was torn at 2nd cycle tensile with 800% strain. Referring to Figure S4 for dynamic mechanical behavior of other PCL‐PUs (ISB‐E and ISB‐2). (e) Versatility of PCL‐PU (ISB‐P) in surgical procedures. The material could be stretched, sutured, bent, twisted, and cut. Scale bar is 10 mm. After suturing to muscle tissue, a stable suture interface between ISB‐P and the tissue was observed in Video S1.

Next, the mechanical properties of PCL‐PU were investigated in tensile mode with a rectangular film (50 × 5 × 0.2 mm), and a stress–strain curve was plotted to visualize the hyperelasticity, as approximately determined by (maximum stress) × (strain at break) values (Figure 1a,b). The new PCL‐PU (ISB‐P and ISB‐E) had higher maximum tensile stress than bare isosorbide‐based PCL‐PU (ISB‐2′), with similar or even higher values than that of PCL and isosorbide‐free PCL‐PU (Figures 1a and S3); specifically, ISB‐P might had hyperelastic properties with 50 MPa maximum stress and ~1100% of strain at break, depicted in the stress–strain curve among values from other PU materials,^21^, ^23^, ^24^, ^25^, ^32^, ^57^, ^58^, ^59^, ^60^ while isosorbide‐free PCL‐PU had poor elasticity. In addition, the maximum stress and strain could be controlled by the ratio between the PCL diol and HDI of ISB‐P with an inverse correlation (Figure 1b).^25^ Due to the similar mechanical property, commercially available high‐molecular weight PCL (*M*_w_ = 224,120), not isosorbide‐free PCL‐PU, was chosen as counterpart of degradable PLC‐PU copolymer for further translational experiments, which have been utilized in various clinical applications including bone regeneration.

To prove super‐elasticity, bulk elastic recovery under tensional force was first determined using ISB‐P as representative (versus PCL) due to its highest maximum tensile strength × strain. A 2D film‐formed PCL‐PU (ISB‐P, 20 × 10 × 0.2 mm) was subjected to 200% tensile strain, and upon release, the length immediately recovered to the original value (105%, ~95% recovery), while that of PCL did not recover. Elastic recovery under compressive stress was also detected using a 3D‐formed cylindrical structure, revealing up to 50% recovery, while PCL showed only up to 20% recovery (Figure 1c). Next, we examined the mechanical behaviors of the PCL‐PU under dynamic stress conditions. The sample was stretched up to 10 cycles with different strain (100%–1100%), respectively. The results demonstrate that the all PCL‐PU tested (including ISB‐P) are highly resilient and flexible enough to maintain 80%–95% of initial (1st) strength against up to maximum tensile strain (100%–1100%) even after 10 times cyclic tensile load (10th) while PCL was torn at 2nd cycle tensile with 800% strain (Figure 1d and S4), supporting the potential applications of PCL‐PU as implantable biomaterials with surgical compatibility against repeated adaptation and the long‐term clinical stability under dynamic force conditions. An example of the surgical compatibility of PCL‐PU (ISB‐P) is illustrated in Figure 1e, revealing elastically stretchable, bendable, twistable without visible deformation and surgical compatibility in terms of cutting and suturing (Video S1).

**FIGURE 2:**
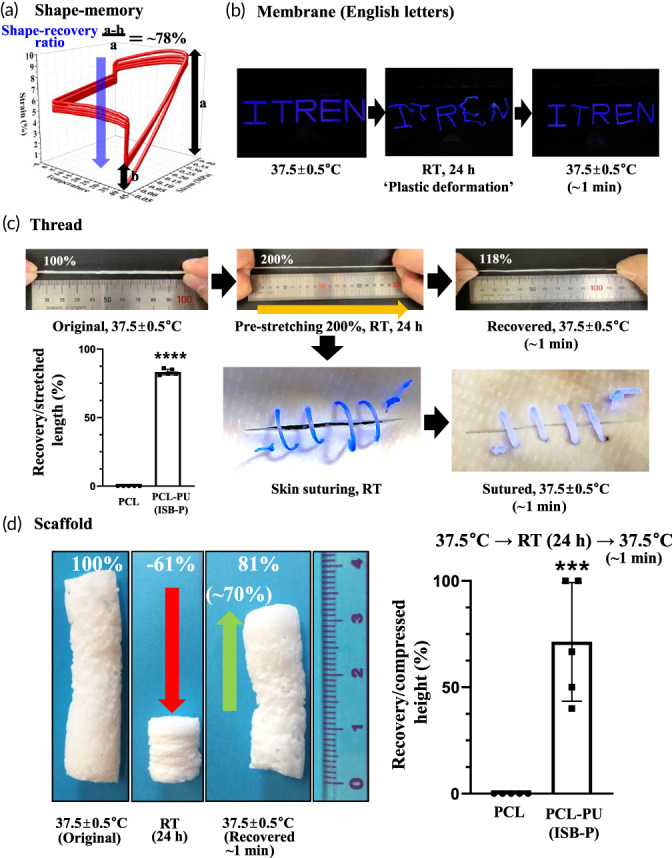
Shape‐memory properties of PCL‐PU with various forms and force directions. (a) Stress–strain response of PCL‐PU (ISB‐P) across a range of temperatures. A total of 4 cycles (40°C ↔ 0°C, 10%) were performed and measured by thermodynamic mechanical analysis, showing ~78% recovery of the original strain from the temporarily deformed (10%) PCL‐PU specimen (ISB‐P) at 40°C, a typical characteristic of shape memory near body temperature. Blue arrow indicating recovery of strain as shape‐memory property. (b) Shape memory of the letters cut from the membrane form: English letters (ITREN) were randomly deformed at room temperature (RT) for 24 h to allow plastic deformation, and shape recovery was detected at 37.5°C. (c) Shape memory of the thread form; thin thread (2 × 100 mm) was stretched at RT up to 200% for 24 h and recovered to its original length (118%, ~82% recovery) at 37.5 ± 0.5°C. Loose suturing using prestretched (~200%) thin thread at RT became tight at 37.5°C (*n* = 5, Video S2). (d) Three‐dimensional scaffolds (40 mm h × 5 mm d) recovered by ~70% at 37.5°C after compression to 39% of the original length (−61%) at RT for 24 h. PCL was used as a negative control without shape‐memory behavior (*n* = 5). ***p < 0.001 and ****p < 0.0001 by *t* test.

Next, shape‐memory characteristics of PCL‐PU were determined by differential scanning calorimetry (DSC). *T*_m_ values, where memory‐based shape‐changes theoretically occur in polymer, was initially determined near body temperature (37.1–38.3°C) for the newly synthesized isosorbides (ISB‐P and ISB‐E) slightly higher than that of bare isosorbide (36.9°C, ISB‐2′) (Figure S5A). When bulk shape memory property under tension was manually performed on film to confirm shape‐memory characteristics briefly, the most recovery property (~82%) was detected in ISB‐P than others (30%–70%), which was selected for further shape‐memory analyses (Figure S5B). Thermodynamic mechanical analysis similarly showed ~78% recovery of the original shape from the temporarily deformed (10%) PCL‐PU specimen (ISB‐P as representative) at 40°C in four independent temperature cycles (40°C ↔ 0°C) (Figure S6), a typical characteristic of shape memory polymer near body temperature, which is comparable to values obtained from other PU‐based shape memory polymers working at 40 or 60°C (32%–95% for shape‐recovery and 40%–95% for shape‐fixity).^22^, ^61^, ^62^, ^63^ These 78% recovery (blue arrow) under tension stress was obtained from combinatory effects of solely shape‐memory, thermal condensation at cooling stage (40 → 0°C) and innate elasticity at unloading status (0.4 → 0 MPa) at low temperature (0°C) during thermodynamic mechanical analysis, indicating the possibility of less shape memory effects in other environments.^64^ To reveal the shape‐memory effect in various forms from different directional forces, three different forms and sizes were fabricated: English capital letters (ITREN) from film membranes (0.2 mm thickness), thin threads (2 × 100 mm), and salt‐leached 3D scaffolds (40 mm h × 5 mm d). After the shape consisting of English letters was memorized at 37.5 ± 0.5°C, the English letters were randomly deformed at room temperature (RT, 20 ± 0.4°C) for 24 h. When the temperature was increased again to 37.5 ± 0.5°C, the original shape of the English letters was recovered from plastically deformed status (Figure 2b). Similarly, when thin thread at RT was stretched up to 200% for 24 h at 37.5 ± 0.5°C, the thread recovered almost to its original length (118%, ~82% recovery) (Figure 2c). When the prestretched (~200%) thread at RT was inserted into pig skin to mimic skin wound suturing and the temperature was increased, the thread tightened to close the wound gap (Video S2). Finally, when the cylindrical 3D scaffold was compressed from the original length down to 39% (−61%) at RT for 24 h to allow plastic deformation and the temperature was increased, the length partially recovered to 81% (~70% of compressed deformation, Figure 2d). However, PCL did not show any shape‐memory property in the all conditions above. Unfortunately, the processability of ISB‐P by 3D printing was limited due to its thermoset property, similar to other PU polymers, and 3D printable, PU based shape‐memory polymer remain elusive. Combined together, regardless of the shape (film, thread, 3D scaffold) and stress direction (random, tension, and compression), shape‐memory characteristics were successfully detected in PCL‐PU (ISB‐P).

### 2.2 Ultra‐cell‐adhesive PCL‐PU biomaterial via Hspa1‐mediated alpha 5 integrin

**FIGURE 3:**
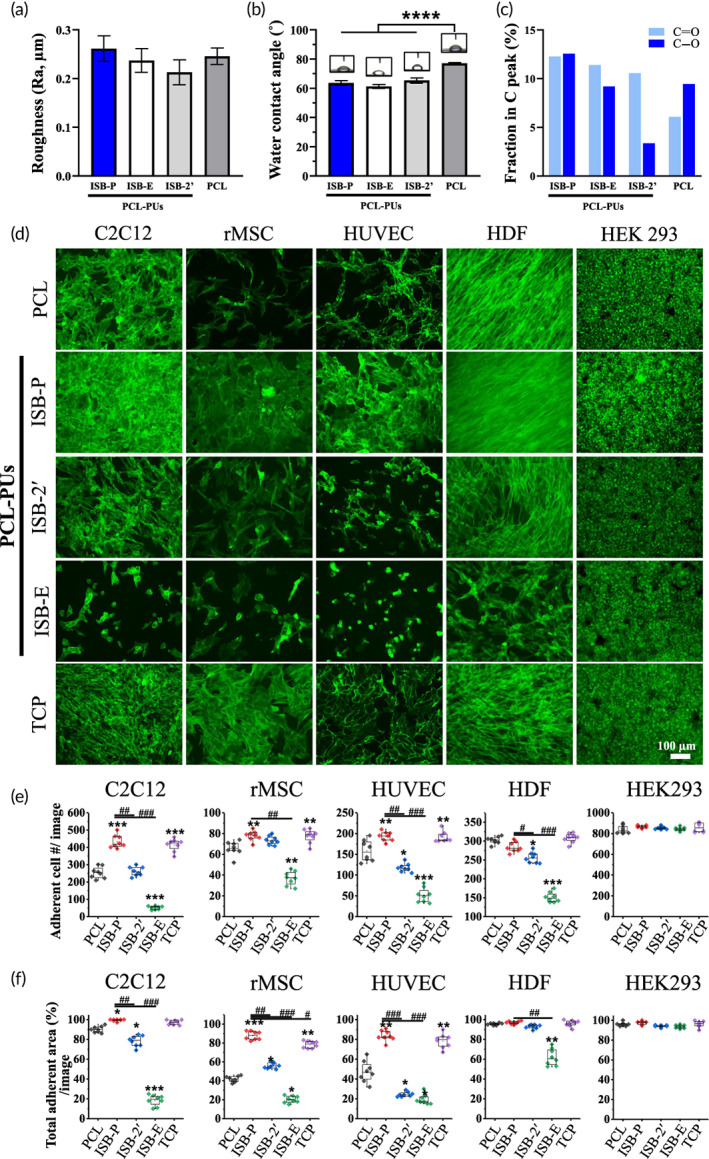
Ultra‐cell‐adhesive property of PCL‐PU (ISB‐P) compared to PCL. (a)–(c) Surface characteristics related to cell adhesion in PCL‐PUs and PCL; (a) roughness (*n* = 3), (b) hydrophilicity by water contact angles (*n* = 4), and (c) polar chemical bonding (C‐O and C=O) fraction in C1s by XPS. PCL‐PUs have lower water contact angle with more polar chemical bonding component than PCL. (d)–(f) Various types of cells were tested to evaluate the cell‐adhesiveness of the as‐cast polymers. C2C12, rMSCs, HUVECs, HDFs, and HEK‐293 cells were selected to represent myoblast cells, mesenchymal stem cells, endothelial cells, fibroblasts, and transfected cells, respectively. ISB‐P generally revealed enhanced adherent cell numbers and total adherent area per image at 24 h after seeding (*n* = 8, normalized by PCL). *p < 0.05, **p < 0.05, ***p < 0.001 and ****p < 0.0001 compared to PCL, ^#^ *p* < 0.05, ^##^ *p* < 0.01, ^###^ *p* < 0.001 compared to ISB‐P, one‐way ANOVA with Dunnett's test. Ultra‐cell‐adhesive properties of ISB‐P compared to that of other PUs in other literatures were additionally depicted in Figure S8.

PCL‐based polymers are well known for having proper cell‐adhesive properties without toxicity compared to other synthetic polymers.^1^, ^65^ As designed with the help of PCL component, PCL‐PUs were assumed to have higher cell‐adhesive properties with great cytocompatibility than other PU based polymers. Thus, the surface characteristics and cell adhesiveness of newly synthesized PCL‐PUs were investigated and compared with those of PCL as a positive control. First, basic surface characteristics related to cell adhesiveness were investigated. SEM and surface roughness analysis were performed, and similar morphology was detected with minimal change of surface roughness for the three types of PCL‐PUs and PCL (Ra = 0.21–0.26 μm) (Figures 3a and S7A). When the water contact angle, one of the basic parameters determining cell adhesiveness by hydrophilicity, was measured, the PCL‐PUs had a lower water contact angle (61°–65°) than PCL (76°–78°), partially supported by prominent hydrophilic chemical bonding fraction (~25%, C‐O and C=O) in PCL‐PUs than PCL (~15%) by XPS^66^ (Figures 3b,c and S7B,C). In summary, PCL‐PUs were found to be more hydrophilic than PCL possibly due to polar chemical‐bonding on surface with comparable roughness, which might have chance to promote cell adhesion than PCL.

Next, the in vitro cell‐adhesive properties of three different PCL‐PUs were investigated in various cell types with TCP and PCL as comparison groups. C2C12, rat mesenchymal stem cells (rMSCs), HUVECs, human dermal fibroblasts (HDFs), and HEK‐293 cells were used as representatives of myoblast cells, MSCs, endothelial cells (blood vessel‐forming cells), fibroblasts and human embryonic kidney cells, which are widely used for plasmid transfection (Figure 3d). Intriguingly, compared of the results in PCL, a significant increase in adherent cell numbers and area at 24 h was observed for all types of cells except HEK‐293 in ISB‐P, similar to tissue culture plate (TCP), while ISB‐E and ISB‐2′ had similar or lower cell adhesiveness (Figure 3d–f). After combining cell adhesion results from other PU‐based materials, which have been reported in literatures in relation to TCP controls, including our previous studies (Figure S8), ISB‐P was highlighted as an ultra‐cell‐adhesive material among PCL‐PUs tested.^21^, ^22^, ^24^, ^25^, ^67^, ^68^ Given its mechanophysical and cell‐adhesive properties, ISB‐P was selected for future in vitro experiments on cell‐adhesive mechanisms and differentiation, along with in vivo studies. In addition, in keeping with the idea that HEK‐293 is known to have relatively low integrin expression than other cell lines^69^, ^70^ and no difference was detected in adhesion among groups only from HEK 293, the key role of integrin is assumed for cells to attach on ISB‐P.

**FIGURE 4:**
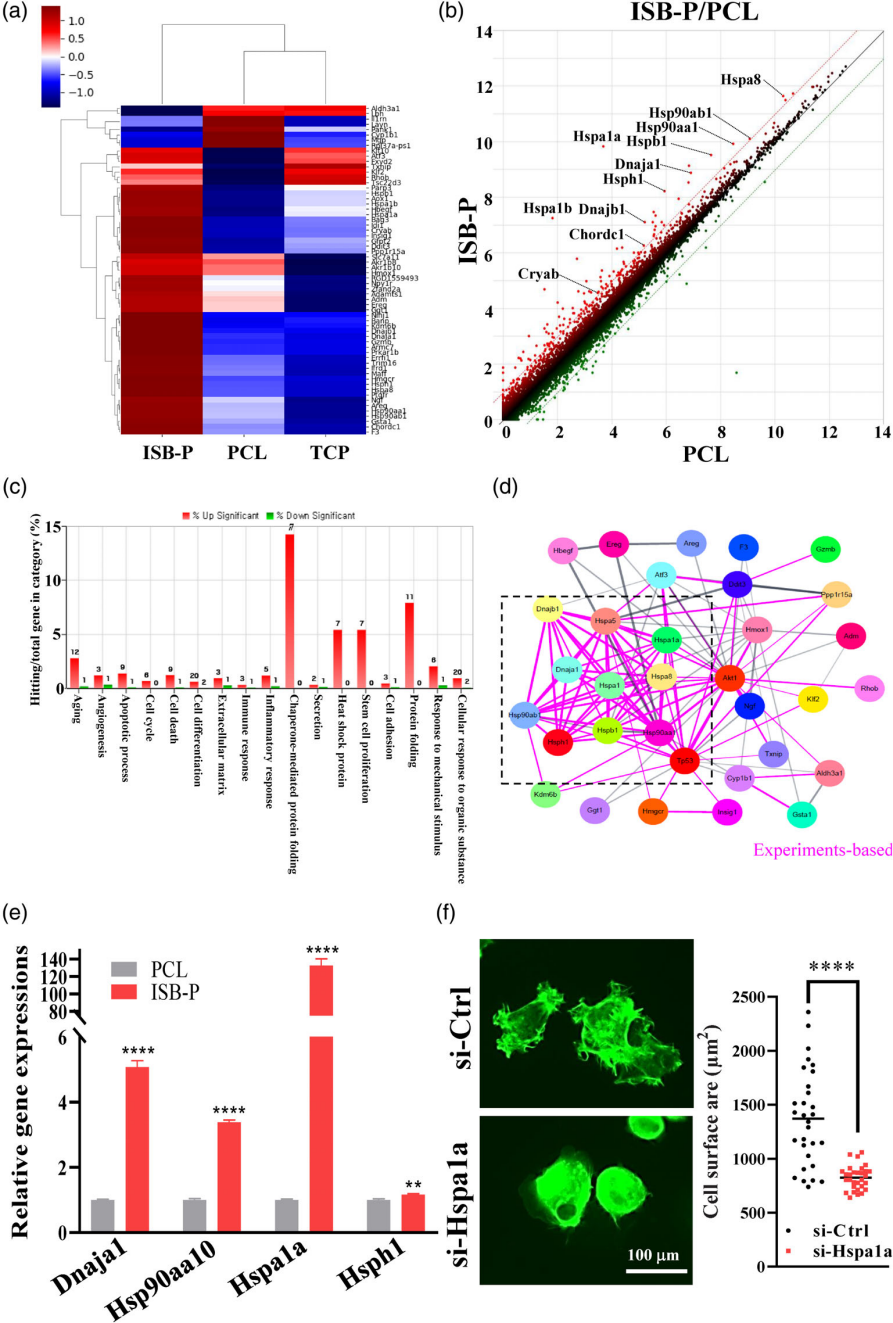
RNA sequence analysis and validation of target gene (Hspa1a) expression to reveal the mechanism of the ultra‐cell‐adhesive property of PCL‐PU (ISB‐P) with mesenchymal stem cells. (a) Heat map of genes with up‐ and downregulated expression in ISB‐P revealed by an average of triplicate RNA sequencing using genes with over 2‐fold changes and normalized values over 16. ISB‐P altered the transcription levels of 62 genes with a 2‐fold increase (54 genes) and a 2‐fold decrease (8 genes) with respect to that in PCL. The results from triplicate experiments between ISB‐P and PCL were depicted in Figure S8 (*n* = 3). (b) Scatter plot of global gene expression profiles of ISB‐P and PCL. RNA expression of all expressed genes in ISB‐P (y‐axis) was plotted against that in PCL (x‐axis) with a log2 scale. A total of 11 genes with upregulated expression related to heat shock proteins and chaperone‐mediated proteins are indicated in the scatter plot. (c) Gene Ontology bar chart of significantly differentially expressed genes between ISB‐P and PCL. Gene categories of 62 genes and the percentage of hit genes/total genes in the category are presented on the x‐axis and y‐axis, respectively. The number on the bar is the number of genes with upregulated (red bars) and downregulated (green bar) expression. The top four categories in terms of a high percentage of genes with upregulated expression were chaperone‐mediated protein folding, protein folding, heat shock protein, and stem cell proliferation. (d) Protein interactions based on a public database of experimental results (Cytoscape, STRING analysis) for the above 11 genes related to heat shock proteins and chaperone‐mediated proteins revealed close relationships among the 11 proteins through the centrally located Hspa1, Hspb1, Hsph1, Hsp90aa1, and Dnaja1. (e) High expression of heat shock protein genes (Dnaja1, Hsp90aa10, Hspa1a, and Hsph1) compared to the PCL control was confirmed by qRT‐PCR (*n* = 3, **p < 0.01, ****p < 0.0001 by *t* test). (f) Representative images of cells transfected with control or Hspa1a siRNA 1 h after cell seeding. Adhesive cell area was quantified by ImageJ and significantly decreased in the Hspa1a knockdown group compared to the control siRNA group (*n* = 30, ****p < 0.0001 by *t* test).

To further explore why ISB‐P has a greater cell‐adhesive effect even than PCL in the biological perspective, RNA‐seq analysis was performed to screen global gene expression. rMSCs were chosen because they are representative biomaterial‐adhesive cells that regenerate tissue. rMSCs were seeded on specimens (ISB‐P, PCL, and TCP), and the differences in the transcriptomes among different specimens were assessed at 4 h after seeding by RNA‐seq analysis (*n* = 3, total 17,147 genes). Distance‐based clustering of three groups of RNA‐seq data showed that ISB‐P was distant from PCL and TCP, which were close together (Figures 4a and S9). ISB‐P altered the transcription level of 62 genes with 2‐fold increases (54 genes) or 2‐fold decreases (8 genes) with respect to PCL (Figure 4b), indicating that the effect of ISB‐P was relatively limited to specific transcripts. ISB‐P/TCP shared ~65% genes with upregulated (38) and downregulated (2) expression with ISB‐P/PCL (Figure S7), indicating that rMSCs at 4 h adhesion on ISB‐P showed unique transcription compared to PCL and TCP (Figure S10). In DAVID analysis of these 62 genes, the one of most enriched Gene Ontology terms, indicating the power of input gene involvement in a specific category after normalization to overall gene expression, was “Chaperone‐mediated protein transport involved in chaperone‐mediated autophagy,” indicating the possible involvement of chaperone‐mediated genes in ISB‐P's cell‐adhesiveness (Figure S11). A graphic chart of the gene hits in certain categories revealed the top four categories in terms of a high percentage of genes with upregulated expression without any downregulated expression, to be chaperone‐mediated protein folding, protein folding, heat shock protein, and stem cell proliferation (Figure 4c). In particular, 11 genes (Chordc1, Cryab, Dnaja1, Dnajb1, Hsp90aa1, Hsp90ab1, Hspa8, Hspa1a, Hspa1b, Hspb1, and Hsph1) that showed upregulated expression in ISB‐P were related to heat shock protein/stem cell proliferation (7 genes; Chordc1, Dnaja1, Dnajb1, Hsp90ab1, Hspa1a, Hspa1b, and Hspa8) or chaperone‐mediated protein (7 genes; Chordc1, Dnajb1, Hspa1a, Hspa1b, Hspa8, Hspb1, and Hsph1), along with protein folding (11 genes above), and are indicated in the scatter plot (Figure 4b). Heat shock protein members were revealed to regulate cell adhesion, spreading and proliferation through their major roles as chaperone proteins to facilitate the proper folding of newly formed or misfolded proteins.^71^, ^72^ Protein interactions based on public databases of experimental results (Cytoscape, STRING analysis) on the above genes revealed close relationships among the 11 proteins through the centrally located Hspa1, Hspb1, Hsph1, Hsp90aa1, and Dnaja1 (Figure 4d). In particular, Akt, a key component in multiple cellular processes, such as protein synthesis, metabolism, cell adhesion and proliferation, and tp53 (p53), a transcription factor gene that acts as a tumor suppressor and is altered in response to cellular stress, were newly detected, both indicating the possible roles of the identified genes in increasing cell adhesion in ISB‐P.^73^, ^74^ To validate the high gene expression and close protein interactions, qPCR analysis was performed using four genes of interest, omitting Hspb1 due to the sequence identity between Hspa1 and Hspb1. Hspa1a had the highest gene expression (~130), consistent with the results of RNA sequencing (fold change 70.2) while other genes (Dnaja1, Hsp90aa10, Hsph1) were highly upregulated (Figure 4e). Hspa1a is one of the members of the heat shock protein 70 family and acts as a chaperone protein, mediating the proper folding of new or damaged proteins and myriad biological processes, including adhesion in various types of cells.^75^, ^76^, ^77^, ^78^, ^79^, ^80^ To confirm the cell‐adhesion role of Hspa1a, control small interfering RNA (siRNA) or si‐Hspa1a was introduced, and a cell adhesion test was performed on ISB‐P. Hspa1a gene knockdown was confirmed by qRT‐PCR after transfection with si‐Hspa1a **(**Figure S12A). After 1 h of cell seeding on ISB‐P, rMSCs transfected with si‐Hspa1a revealed a significant decrease in cell‐adhesion area with round shape compared with the control siRNA‐treated group, which showed strong focal adhesion sites throughout a broad area (Figure 4f). This result indicates that Hspa1a is important in mediating the cell adhesion and spreading of rMSCs on ISB‐P.

**FIGURE 5:**
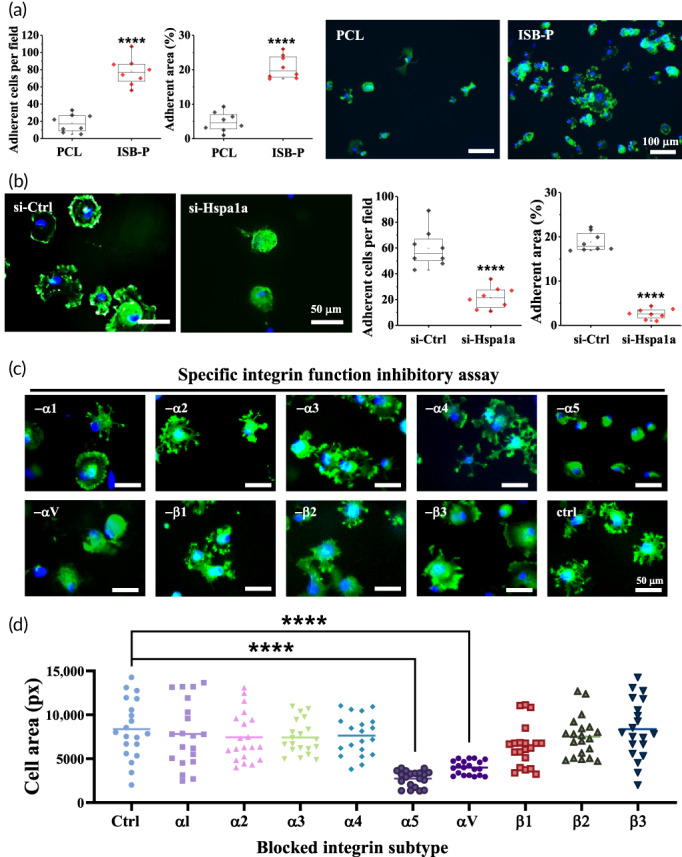
Hspa1a mediated integrin α5 and αν expression as a PCL‐PU (ISB‐P) cell‐adhesive mechanism with human mesenchymal stem cells. (a) Cell adhesion number, area, and representative images stained with DAPI (blue) and phalloidin (green) for the nuclei and F‐Actin of hMSCs on PCL and ISB‐P at 1 h after seeding (*n* = 8). (b) hMSCs treated with si‐Hapa1a for Hspa1a gene knockdown showed lower number and area of adherent cells than the si‐Ctrl group (*n* = 8). (c)–(d) Integrin functional blocking assays involving cell adhesion on PCL‐PU (ISB‐P). Each integrin subunit was blocked through preincubation with integrin blocking antibody for 15 min at 37°C. Preincubated cells were seeded and cultured onto ISB‐P for 1 h at 37°C. Adhered cells were stained with phalloidin (green) and DAPI (blue) and quantified by ImageJ (*n* = 20). The results indicated that integrin α5 and αν are involved in the adhesion of hMSCs to ISB‐P. ***p < 0.0001, *n* = 8 or 20 by *t* test or one‐way ANOVA with Dunnett's test.

Next, the ultra‐cell‐adhesive property of ISB‐P was confirmed with a human stem cell lineage, human MSCs (hMSCs), which were 4 times more highly adherent on ISB‐P than on PCL (Figure 5a). As in rMSCs, Hspa1a knockdown on hMSCs (Figure S12B) decreased the adherent cell number per image and area (%) to 15%–30% of that with the control siRNA (Figure 5b). To further investigate the specific involvement of adhesion receptors (integrins) in Hspa1a‐mediated cell attachment on ISB‐P, a functional integrin blocking assay was performed with 15 min of preincubation condition. The results showed a significant inhibitory effect on cell adhesion under integrin *α5* or *αν* blocking conditions, indicating a pivotal role of integrin *α5* and *αν* in stem cell early attachment on ISB‐P regardless of integrin beta subunit (Figure 5c,d). Integrin *α* subunits are more dynamically changed to component of extracellular matrix by physical recognition than integrin *β* subunits involving physically linking to actin‐filament.^81^, ^82^ However, the gene expression level of integrin *α5* and *αν* was not changed in Hspa1a knockdown stem cells (Figure S13), supporting the post‐transcriptional role of Hsp1a for stem cell adhesion as a chaperone protein, regulating protein folding for activation. Both *α5* and *αν* integrin are well addressed integrin subunit for cell adhesion^83^, ^84^; *α5* integrin is known to play a key role in stem cell attachment on fibronectin‐coated surfaces (also called fibronectin receptor along with *β*1 integrin) while *αν* integrin, called vitronectin receptor along with *β*3 integrin, is involved in vitronectin and fibronectin recognition. To decouple the total amount of adsorbed fibronectin, one of the key cell adhesion molecules governing cell‐ECM sensing, adhesion and differentiation,^85^ from the enhanced cell attachment, the initial adsorption amount of fibronectin within 1 h was investigated using rhodamine‐conjugated fibronectin. Similar fibronectin adsorption amounts and supraphysiologically high stiffness (30–50 MPa) than pre‐mineralized extracellular matrix were detected in ISB‐P and PCL (Figure S14), reinforcing the involvement of biological factors, here Hsp1a‐induced integrin α5 and *αν* functional activity, in enhancing stem cell adhesion on ISB‐P.^86^ Taken together, these results revealed that the integrin *α5* and *αν* subunit functions as major surface receptors for ISB‐P in stem cells possibly by the activation of Hspa1a in a special biological signaling pathway,^87^, ^88^, ^89^ not by physical factors such as surface roughness and adhesive protein adsorption. Further study is necessary to investigate whether the activation of Hspa1a genes by ISB‐P to regulate cell adhesion is unique among PU‐PCL shape‐memory polymers and what are the key chemical structures to tune Hspa1a genes.

### 2.3 Differentiation conductive PCL‐PU biomaterial

Bare PCL showed lower initial cell attachment at 24 h after seeding than ISB‐P (Figure 3d–f). To minimize the surface characteristics while improving cell adhesion capability, PCL film was treated by non‐thermal oxygen plasma (PCL@OP) (Figure S15A,B). PCL@OP showed higher hydrophilicity with enhanced C‐O and C=O bonding structure than bare PCL while the surface roughness did not change. The minimal effect of non‐thermal oxygen plasma to surface roughness and bulk molecular structures has been reported under defined condition previously, which condition was selected here.^66^, ^90^, ^91^ The initial cell attachment at 24 h of PCL, PCL@OP, and ISB‐P were compared in rMSC and C2C12, displaying increase of cell adhesion property of PCL@OP similar to ISB‐P (Figure S15C). Thus, as a cell‐adhesiveness counterpart of ISB‐P, PCL@OP is selected for all further experiments. Cell differentiation capacity on ISB‐P was investigated for muscle and bone, which are the most abundant target tissues for regeneration in the biomedical field. First, to investigate the possibility as tissue‐engineering platform, C2C12 myoblast cells were cultured on ISB‐P. C2C12 myoblast cells matured better on ISB‐P than PCL@OP after 7 days based on the levels of myosin heavy chain (MHC)‐stained myotubes and their widths as muscle cell differentiation markers (Figure S16A). Next, to utilize the elasticity of ISB‐P, stretching culture could be applied to achieve biomimetically aligned skeletal muscle tissues, as skeletal muscle fiber construction for tissue‐engineered implants requires the assembly of unidirectionally aligned juxtaposed myotubes.^92^, ^93^ C2C12 cells were seeded on unidirectionally electrospun ISB‐P nanofibers (700–1300 nm), indicating 90% alignment along the fiber direction (Figure S16B), and stretching culture conditions (4 s stretching (10%) and 6 s rest per cycle, 1 h per day) were then applied.^94^ The number and width of MHC‐stained cells increased at the early differentiation time point (day 4), revealing possibility of ISB‐P for accelerated muscle tissue engineering under dynamic tensional culture conditions due to elasticity (Figure S16C).

**FIGURE 6:**
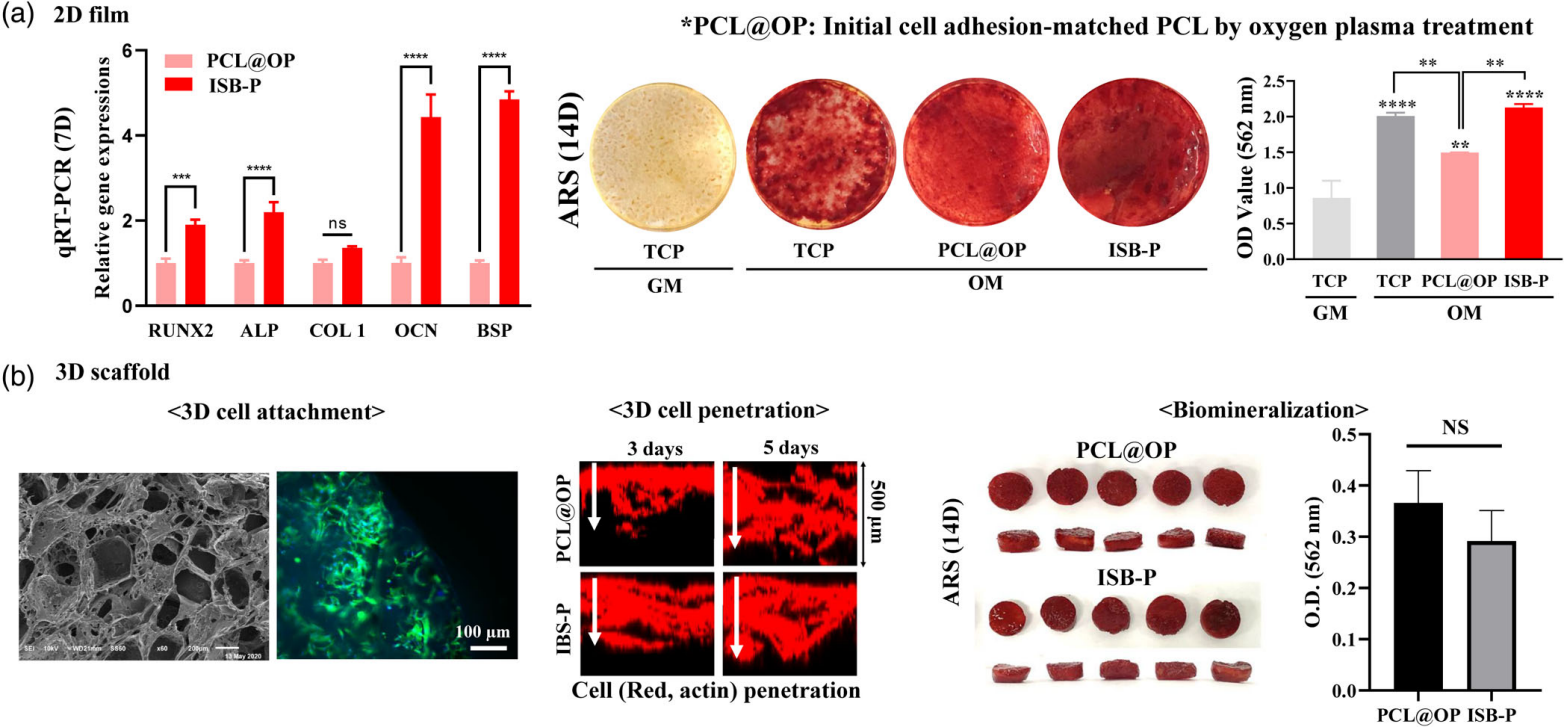
Osteogenic differentiation‐conductive properties of PCL‐PU (ISB‐P) in 2D and 3D culture conditions. (a) Osteogenic differentiation of rMSCs on 2D film. Initial cell adhesion was matched in PCL by oxygen plasma treatment (PCL@OP) compared to ISB‐P, osteogenic potential of ISB‐P was evaluated. Osteogenic gene (RUNX2, ALP, Col1, OCN, and BSP) expression was analyzed by qRT‐PCR on day 7, and biomineralization was evaluated by ARS staining on day 14. The optical density (OD), representing biomineralization, was quantified at 563 nm. ISB‐P showed a slight increase in osteogenic differentiation compared to cell adhesion‐matched PCL@OP, similar to TCP (*n* = 4). (b) The interconnected porous (10–500 μm) structure of the ISB‐P 3D scaffold, made by the salt‐leaching method, was revealed by SEM, and the morphology of rMSCs adhered to 3D scaffold pores at 24 h was visualized by fluorescence microscopy (actin for green and nucleus for blue). Cell penetration inside 3D scaffolds was observed on days 3 and 5, and similar cell penetration depths were observed. ARS‐stained images of rMSCs cultured for 14 days in 3D scaffolds in osteogenic induction medium and their quantification (*n* = 5). ns (not significant), **p < 0.01, ***p < 0.01, ****p < 0.0001, by *t* test or one‐way ANOVA with Dunnett's tests.

To investigate the eligibility of PCL‐PU (ISB‐P) as implanted biomaterials for tissue regeneration, an osteogenic capacity was revealed as representative. In vitro osteogenesis study using MSCs cultured on 2D film structure revealed that ISB‐P had higher expression of early and late osteogenic genes (RUNX2, ALP, BSP, and OCN) than PCL@OP with enhanced biomineralization, as revealed by qPCR and Alizarin red S (ARS) staining (Figure 6a). To assess the 3D‐bone forming ability, 3D ISB‐P scaffold with interconnected porous was fabricated by the salt‐leaching method with pore sizes (50–500 μm) similar to that of PCL (Figure S17). When the cell adhesion and mineralization of MSCs were tested, cell adhesion was well recognized even inside pores, and penetration of cells to inner scaffold structures through interconnected pores over 5 days of culture was similarly visualized (Figure 6b). Biomineralization at 14 days of differentiation was comparably detected between groups unlike the data from 2D film. The discrepancy between 2D and 3D culture data might come from bulk elasticity and molecules diffusion.^95^, ^96^, ^97^

In summary, myoblast cells and osteoprogenitors differentiation on ISB‐P was confirmed with 2D film, pseudo3D electrospun or 3D‐porous structures even under tensile stress (for muscle differentiation), showing the versatility of ISB‐P for tissue regeneration due to its elasticity, cell‐adhesive and differentiation‐conductive properties with appropriate cytocompatibility for long‐term culture (up to 14 days). Although the mechanism study might be needed to understand the significant increase of osteogenic differentiation in ISB‐P (even though it was observed only in 2D films), the engagement of heat shock proteins upregulated in ISB‐P during cell adhesion is suggested as one of the possible biological machinery. Because there was no significant difference in surface characteristics, including roughness, hydrophilicity, and chemical structure, between ISB‐P and PCL@OP, while, HSP gene levels were higher in ISB‐P. In various literatures, heat shock proteins have been reported to promote osteogenic differentiation via activation of Wnt/b‐catenin signaling (HSPA1A‐HSP70 family^79^), ERK pathway (HSP70,^98^ HSPB7^99^), and several strategies have been tried for decades to enhance HSP level through application of physical stress such as periodic heat shock (for HSP70)^100^ and ultrasound (for HSP70 and 90).^101^ Overall, the osteogenic differentiation might be promoted via upregulation of osteogenic signaling, which was enhanced by continuously higher heat shock proteins levels on ISB‐P from initial cell attachment.

### 2.4 In vivo biocompatibility and bone induction of the PCL‐PU biomaterial

**FIGURE 7:**
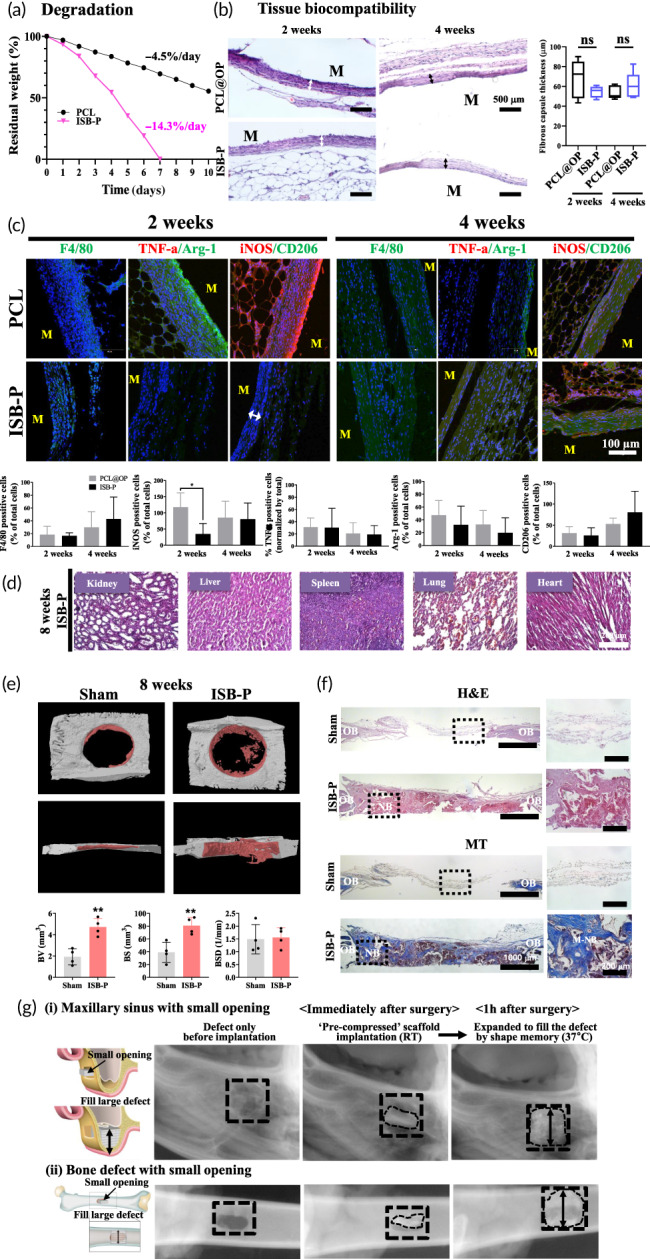
In vitro and in vivo biocompatibility and bone induction ability of PCL‐PU (ISB‐P). (a) Enzymatic degradation of ISB‐P using NaOH (5 M) at 37°C in dynamic conditions at 120 rpm for 53 days. (b) H&E staining 2 and 4 weeks after subcutaneous implantation. The fibrous layer thickness and inflammatory scores surrounding the materials were measured to analyze the tissue compatibility. ISB‐P showed similar fibrous thickness than PCL@OP with mild inflammation scores, indicating biocompatibility (*n* = 5). (c) Immunohistochemistry analysis of the tissue immune response 2 and 4 weeks after subcutaneous implantation of PCL‐PU, semiquantifying pan‐ (F4/80), inflammatory (iNOS and TNF‐a) or anti‐inflammatory (CD206 and Arg1) macrophages (*n* = 5). DAPI‐stained nuclei (blue). Scale bars represent 200 mm. (d) In vivo toxicity to main organs (kidney, liver, spleen, lung, and heart) in subcutaneously implanted ISB‐P groups at 8 weeks (*n* = 3). The organ sections were stained with H&E and no significant systemic toxicity was observed. (e) and (f) Bone regeneration analysis by micro‐CT and histology in rat calvarial defects 8 weeks after implantation of the ISB‐P 3D scaffold (*n* = 4). Micro‐CT images showed enhanced mineralization in ISB‐P by quantification of bone volume (BV), surface (BS), and surface density (BSD). H&E and MT staining confirmed collagen (blue and pink) and new bone formation (dark pink and navy) in the ISB‐P group. (g) Shape‐memory characteristics of ISB‐P in vivo (rabbit); (i) maxillary sinus bone and (ii) small‐opening bone defects (*n* = 3). A 3D scaffold (consisting of ISB‐P) compressed at room temperature (RT, ~30% from original height) was transplanted into the regenerative site of rabbits through a small defect opening. After 1 h in the rabbit body (37°C), the height of 3D scaffolds coated with x‐ray‐detectable chemicals recovered to the original value in both areas, as detected by x‐ray imaging. ns (not significant), *p < 0.05, **p < 0.01, ***p < 0.001, ****p < 0.0001, by *t* test.

Degradation study using base chemical (NaOH, 5 M), hydrolyzing esters by OH radical into a salt and alcohol, was performed to reveal degradation ability. It showed a degradation rate of ~14.3 wt%/day from ISB‐P, much faster than that of PCL (~4.5 wt%/day) along with other PCL‐PUs (7–12.5 wt%/day, Figures 7a and S18A) due to as‐given hydrophilic characteristics, increasing the water penetration into the matrix and give increased rates of hydrolysis of the ester and urethane bonds.^25^ In challenge with enzymatic degradation using esterase (2 mg/ml), enzymatically splitting esters into an acid, and alcohol,^102^ ISB‐P displayed faster degradation (0.8 wt%/day) while PCL rarely degraded along with other literatures.^103^, ^104^ With raised concerns about biocompatibility from byproducts during degradation of the polymer, the initial innate immune response of biodegradable PCL‐PU was briefly investigated. Human monocytes (THP1) were differentiated into macrophages by PMA (phorbol 12‐myristate 13‐acetate, 50 ng/ml) and seeded on PCL@OP and ISB‐P. ISB‐P did not induce higher M1‐like macrophage inflammatory gene (TNF‐a, IL‐1b, and IL‐6) expression compared to PCL@OP after 24 h of adhesion (Figure S18B). Next, in vivo tissue biocompatibility was tested in a rat subcutaneous implantation model. After 2 and 4 weeks of implantation, hematoxylin and eosin (H&E)‐stained images revealed similarly small thickness of the fibrous layer, resulting in comparable compatibility in ISB‐P with respect to that of PCL@OP (Figures 7b and S19). Immunostaining results supported the above histological features with similar pan‐macrophage (F4/80), inflammatory (iNOS), and anti‐inflammatory (CD206)+ cells in both groups at 2 and 4 weeks implantation (Figure 7c). Further investigation for a possible long‐term local inflammatory reaction from highly enzymatic degradable ISB‐P is essential for clinical application. With regard to the in vivo toxicity of ISB‐P, the possible systemic toxicity in major organs (kidney, liver, spleen, lung and heart) was analyzed after subcutaneous implantation for 8 weeks, although previous literature reported little or negligible organ toxicity of PU or PCL‐based polymers.^105^, ^106^ Histological observation of the tissue samples confirmed the nondetectable organ toxicity of the ISB‐P‐implanted species (Figure 7d).

Following the above in vitro immune response and in vivo biocompatibility studies showing comparable biocompatibility between FDA‐approved polymer, PCL, and ISB‐P, the therapeutic tissue regenerative effect was investigated using a rat calvarial critical bone defect model (∮ = 5 mm) with a salt‐leached 3D scaffold. After 8 weeks of implantation, μCT 3D images were reconstructed and revealed a significant increase in neobone formation (dark pink colored) within the defect region in the ISB‐P group, although the healing process was not complete due to the relatively short time period; when quantitated, neobone formation in the ISB‐P group was significantly higher in terms of bone volume and surface area but similar in bone density to that of the sham group (Figure 7e). Based on μCT 3D images, ISB‐P had comparable bone formation to PCL@OP (Figure S20). Histological observation of the H&E‐ and Masson's trichrome (MT)‐stained samples revealed newly formed bone and osteoids in the healing areas without obvious inflammatory cells near the 3D scaffold (Figure 7f). Highly magnified images (block dot rectangle) showed comparable matured (strong blue color in MT stained image) bone filling in the pores, in contrast to no bone formation in the sham operation defects. The in vivo results together showed that ISB‐P was highly effective, even comparable to PCL, in accelerating early neobone regeneration, suggesting potential applications as an implantable biomaterial for bone repair and regeneration.

To investigate the possible clinical applicability of ISB‐P based on its shape memory characteristics, two in vivo clinical models were suggested: maxillary sinus bone lift and a bone defect with a small outer exposure but a large inner space to fill in, possibly caused by a bullet or sharp weapon. After memorization of the original shape at 37°C, the ISB‐P scaffold, coated with radiopacifier (iodoform) for visualization by x‐ray, was compressed at RT (~30% from the original height) and inserted into the rabbit maxillary sinus and tibia bone. Comparison of x‐ray images immediately after implantation and 60 min later in the rabbit body (37°C) showed that the ISB‐P scaffold was enlarged compared to the condensed structure, which was a unique characteristic of the shape‐memory polymer, unlike the PCL derivatives (Figure 7g). Although the bone regenerative potential of the recovered ISB‐P was not directly investigated, it was assumed to have similar bone‐forming ability to that in a rat calvarial defect model, due to identical chemical structure before and after the shape change. The in vitro and in vivo results together showed that ISB‐P accelerated regeneration of tissue, including bone, which might result from the collective effects of ultra‐cell adhesiveness, biocompatibility without distinct immune response, target‐tissue conductivity, and shape‐memory properties in various forms, indicating the potential applications of ISB‐P in tissue‐regenerative implantable biomaterials.

## 3 CONCLUSION

**FIGURE 8:**
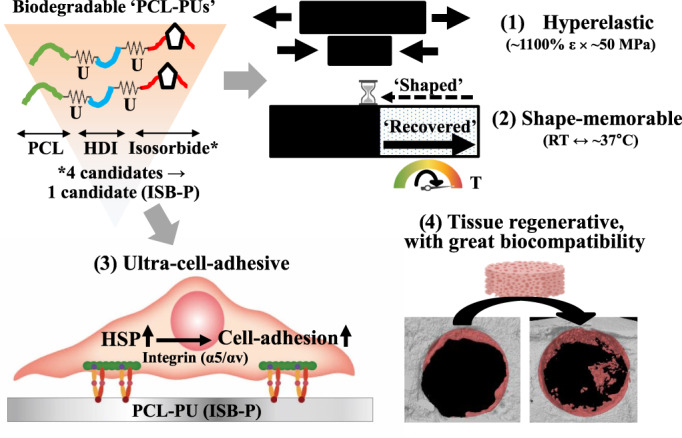
Summary of optimal PCL‐PU (ISB‐P) characteristics for tissue regeneration. Biodegradable PCL‐PUs were newly synthesized with PCL, HDI and isosorbide or its derivatives. From four candidates, ISB‐P was selected as optimal polymer in tissue regeneration or engineering. Currently optimized PCL‐PU (ISB‐P) is hyperelastic (~1100% strain × ~50 MPa), shape‐memorable (room temperature (RT) ↔ ~37°C), ultra‐cell‐adhesive (better than bare PCL) via HSP mediated integrin activation, and tissue regenerative with great biocompatibility against tissue and major organs. HDI, 1,6‐hexamethylene diisocyanate; HSP, heat shock protein; PCL, polycaprolactone; RT, room temperature; U, urethane group.

Here, the currently exploited PCL‐PU (especially ISB‐P) were synthesized for the fabrication of a biocompatible, and multifunctional (hyperelastic, shape‐memorable, ultra‐cell‐adhesive, regenerative, and degradable) tissue‐regenerative biomaterial (Figure 8). The unique combination of notable mechanical performance, shape‐memory effects, and ultra‐cell‐adhesive properties make appropriate forms (e.g., patch, thread, electrospun, 3D scaffold) of the engineered material suitable as a regenerative substrate for various tissues (e.g., bone, muscle, and skin) in the biomedical field. Cell adhesion mechanism of ISB‐P was distinctly revealed as heat shock protein‐mediated integrin α5 and αV activation, leading to further development of newly synthesized, tailored polymer for enhancing specific biological behavior. The biocompatibility, enhanced biodegradability, tissue‐regenerative ability, and in vivo shape memorability of the material can be leveraged to replace PCL polymers, especially for the fabrication of body temperature‐responsive implantable biomaterials to regenerate defects in tissues with limited anatomical accessibility for filling the whole defect, especially for minimally invasive surgery.

## 4 EXPERIMENTAL SECTION

### 4.1 Synthesis of PCL‐PU

PU series materials were synthesized by a one‐step polymerization reaction. PCL diol (*M*_w_ = 2000 g mol^−1^, Sigma‐Aldrich, St. Louis, MO) and isosorbide derivatives (ethoxylated isosorbide, propoxylated isosorbide, bare isosorbide, Samyang, 99.9% purity) were dried at 60 °C for 12 h under vacuum before using and HDI was used as supplied. For ISB‐containing PUs, PCL diol and isosorbide derivatives were placed in a four‐necked round bottom flask (500 ml) equipped with a condenser, thermometer and mechanical stirrer, and heated at 80°C for 30 min under a nitrogen purge. After the mixture was melted thoroughly, HDI (>98%, TCI) was added and vigorously stirred for 5 min under a nitrogen purge. The reaction mixture was poured into a polytetrafluoroethylene plate (PTFE) and polymerized for 8 h in a 120°C oven.^21^, ^24^ After the synthesized PU was dissolved in *N*,*N*‐dimethylformamide (DMF, >99%, Duksan reagent) at 20 wt%, it was precipitated in isopropyl alcohol (IPA, 99%). The precipitated PU was fully vacuum‐dried at 40°C for 48 h to remove the DMF and isopropyl alcohol solvent. The ISB‐free material was synthesized in the same manner as above using only PCL diol and HDI.

### 4.2 Fabrication of films, nanofibers, and 3D scaffolds

The PUs were dissolved in N,N‐dimethyl‐formamide at 20 wt% and stirred at room temperature for 24 h. A 20 wt% polymer solution was poured into a PTFE plate and dried in an oven at 60°C after horizontal alignment. The resulting films were placed in a 60°C oven for 12 h, then vacuum‐dried at room temperature for 48 h to remove the remaining solvent and water. The average thickness of the films was 0.2 ± 0.02 mm. The electrospinning system included a high‐voltage supply (HV30, NanoNC, Seoul, Korea), and a syringe pump (KDS100, NanoNC, Seoul, Korea) maintained the speed of spinning at 0.1 ml/h. The voltage was approximately 17 kV. The ISB‐P solution was loaded into a plastic syringe (10 ml) with a 21 gauge stainless steel needle. Nanofibers were obtained by a collector (DC90, NanoNC) wrapped in aluminum foil located 15 cm away. Three‐dimensional scaffolds were fabricated by solvent‐casting and salt‐leaching techniques. PCL and PCL/PU particles were dissolved at 20 wt% in a cosolvent of 1,4‐dioxane/chloroform at a 4/1 ratio. The polymer solution was poured into a cylindrical plastic mold containing sodium chloride (NaCl) particles with sizes in the range of 325–425 μm and 45–100 at a 7/3 weight ratio. The samples were frozen at −80°C overnight and freeze‐dried for 3 days. The lyophilized samples were cut to 10 mm diameter with 40 mm height, soaked in distilled water for 12 h to remove salts, and dried.

### 4.3 Chemical characteristics

The ^1^H NMR spectra for the synthesized PU as a 5% (w/v) polymer solution in CDCl_3_ were recorded by a Bruker Avance 500 spectrometer. The number average molecular weight (*M*_n_), weight average molecular weight (*M*_w_), and polydispersity index (PDI) of each PU were measured by gel permeation chromatography using ACQUITY Advanced Polymer Chromatography System (Waters Corporation, Milford, MA) equipped with a refractive index (RI) detector. Tetrahydrofuran (THF) was used as the eluent at a flow rate of 0.1 ml/min. Polystyrene (*M*_w_ = 47,200, 129,000, and 264,000) was used as the standard. FTIR spectra were obtained using a Varian 640‐IR in the range of 4000 to 800 cm^−1^. DSC (Exstar 7020) was used for thermal analyses of the samples. Specimens (~20 mg) were sealed in a DSC aluminum pan, placed in the calorimeter, cooled to −70°C, and then heated to 200°C at a rate of 10°C/min under a nitrogen atmosphere. X‐ray photoelectron spectroscopy (XPS) characterization was performed using x‐ray photoelectron spectroscopy (XPS; MultiLab ESCA2000, VG Scientific, UK) with monochromatic Al Kα x‐ray radiation (*hν* = 1486.6 eV) at 120 W. Oxygen plasma treated PCL film was immediately investigated by XPS.

### 4.4 Physical properties

The surface roughness was measured by a surface roughness meter (SJ‐400, Mitutoyo, Kawasaki, Japan). The measurement length, cutoff values, and scan speed were 4 mm, 0.8 mm and 0.5 mm/s, respectively. The average value of three measurements was determined as the value for each specimen. Scanning electron microscopy (JSM‐5410LV) photographs were obtained to visualize the surface morphology at a magnification of 50.00 kX. The contact angle (Phoenix 300) was used to measure the wettability of the polymer surface. Ultra‐pure water droplets (approximately 5 μl) were dropped onto the sample film at intervals of 1 s using a manual syringe to measure the contact angle. Oxygen plasma treated PCL film was immediately investigated by above experiments.

### 4.5 Mechanical properties

Mechanical tensile tests were performed with an Instron universal testing machine (Model 3344, Instron Engineering Corp., Canton, MA). Dumbbell specimens (films) with a thickness of 0.2 mm, a width of 5 mm, and a length of 50 mm were prepared. The samples were measured at a crosshead speed of 10 mm/min at room temperature. The reported results are the mean values for 5 replicates per experiment. For dynamic mechanical behavior test, cyclic tensile loading was applied to the sample for up to 10 cycles with different strain (100%–1100%) individually, and the effect of repeated elastic behavior on tensile stress was observed for 10 cycles. For testing elastic recovery under manual tensile force, both PCL and ISB‐P films were cut into 10 mm width × 20 mm length × 0.2 mm thickness. Then, the original width was stretched by a factor of 2, to 40 mm at RT, and the immediate recovery rate was determined after normalizing to the original width. For the mechanical compressive test, 3D scaffold samples (8 mm height × 5 mm diameter) with porosity were compressed at RT to 42%–43% and checked the immediate recovery rate through photos after releasing. Surgical compatibility test was performed using above rectangular shaped specimen with different thickness (10 × 20 × 0.2–0.5 mm).

### 4.6 Shape‐memory test

ISB‐P was dissolved in N,N‐dimethyl‐formamide at 20 wt%, stirred at room temperature for 24 h, and dried in the oven with appropriate polytetrafluoroethylene mold (0.2 mm × 5.0 mm × 10 mm) at 60°C. The shape‐memory test was initially performed using a dynamic mechanical analysis (Q800) instrument. (1) Samples were kept at 40°C for 10 min. (2) The initial strain (*ε*_1_) was measured after elongation at a rate of 0.5 N/min at a force of 1 N. (3) The sample was cooled to 0°C at a rate of 5°C/min at constant force and then held for 1 h. (4) The force was further reduced to 0 and held for 20 min, and the strain was recorded (*ε*_2_). (5) The specimen was heated again to 40°C at a rate of 5°C/min and held for 1 h, and the residual strain was recorded (*ε*_3_). The shape‐fixity ratio (Rf) and shape‐recovery ratio (Rr) were calculated using the following equations. *R*_f_ = *ε*_2_/*ε*_1_ and *R*_r_ = (*ε*_1_ − *ε*_3_)/*ε*_1_.^107^ This process was carried out for a total of 4 cycles. The English capital letters I, T, R, E, and N were manufactured from a mold. The manufactured capital letters were fixed at approximately 37.5°C ± 0.5, artificially deformed at room temperature to have permanent deformation, restored to 37.5 ± 0.5°C, and confirmed by photography. A line was manufactured on a polytetrafluoroethylene plate 2 mm wide and 100 mm long. After the length of the line was determined at approximately 37.5 ± 0.5°C, it was stretched to 200 mm at room temperature for 24 h to have permanent deformation (~200%). Then, it was restored at 37.5 ± 0.5°C to determine recovery length, as confirmed in a photo. In addition, a cylindrical porous scaffold with a diameter of 10 mm and a height of 40 mm was compressed at room temperature and fixed for 24 h to have permanent deformation (~39% deformation). The structure was placed back at 37.5 ± 0.5°C and photographed.

### 4.7 Degradation test

All types of films were punched with a diameter of 16 mm and a thickness of 0.18 ± 0.01 mm, immersed in 5 ml of sodium hydroxide (5 M NaOH) or esterase (E3019‐29, Sigma Aldrich, 24 units/mg) solution (2 mg/ml in phosphate buffered saline, PBS) in individual tubes, and incubated at 120 rpm and 37°C. The films were washed with deionized water, dried, weighed, and placed in fresh solution (NaOH every day for 10 days and esterase every 3 days for 53 days).

### 4.8 Cell culture

C2C12 (CRL‐1772), HUVECs (CRL‐1730), HEK‐293 (CRL‐1573), and hMSCs (PCS‐​500‐012) were purchased from the American Type Culture Collection (ATCC, Manassas, VA). HDFs (PH10605A) were obtained from Genlantis (San Diego, CA). hMSCs were cultured in alpha MEM containing 10% fetal bovine serum (FBS, 35‐015‐CV, Corning, NY), 1% penicillin–streptomycin (P/S, 15140–122, Gibco, Grand Island, NY), and 2 mM GlutaMAX (35050‐061, Gibco). HUVECs were maintained in vascular cell basal medium (PCS‐100‐030, ATCC) supplemented with Endothelial Cell Growth Kit‐VEGF (PCS‐100‐041, ACTT). Other cell types, such as C2C12 (DMEM, Welgene, Inc., Deagu, Korea), HEK (DMEM, Welgene), and HDF (DMEM, Welgene), were cultured in appropriate cell culture media supplemented with 10% FBS and 1% P/S.

### 4.9 Primary cell culture of rMSCs

rMSCs were isolated from a 6‐week‐old male Sprague–Dawley rat (DBL Co., Ltd., South Korea). The rat was euthanized by CO_2_ inhalation, then sacrificed by cervical dislocation. An incision was made in the ankle joint and side of the hind limbs, and the skin was removed using forceps. The hind limbs were disconnected from the trunk by scissors and placed in PBS after cutting under the ankle region. Muscles and connective tissue were removed from the femurs and tibias, and these bones were used for further step. Then, the bone marrow was separated from the tibias and femurs of the rats using centrifugation in 1.5 ml microcentrifuge tubes at a speed of 10,000*g* for 1 min. The separated bone marrow was suspended in growth medium (alpha MEM supplemented with 10% FBS and 1% P/S) and passed through a 70 μm cell strainer (352,350, Falcon, CA) to remove bone debris and blood aggregates. The filtered suspension was centrifuged at 2000 rpm for 5 min. The supernatant was aspirated, and the cell pellet was suspended in growth medium, seeded in a 100 mm culture dish, and cultured at 37°C in a humidified atmosphere of 5% CO_2_. The medium was changed every 2 days. All protocols involving animals were conducted according to the standards of IRB in the Dankook University regulations.

### 4.10 Cell adhesion assay

A cell adhesion study was performed on 6 mm diameter films of each material sterilized by EO gas. Cells were seeded at a density of 6.25 × 10^4^ cells/cm^2^ in 100 μl of culture medium and incubated at 37°C in a humidified atmosphere of 5% CO_2_ for 1 (cell‐adhesive mechanism study) or 24 h (cell‐adhesive property test). Then, the cells were fixed with 4% PFA, permeabilized with 0.5% Triton X‐100 for 10 min and incubated for 30 min with Alexa Fluor™ 546 Phalloidin (A22283, Invitrogen, Carlsbad, CA) or Alexa Fluor™ 488 Phalloidin (A12379) and 4′,6‐diamidino‐2‐phenylindole dihydrochloride (DAPI, D1306, Thermo Fisher Scientific) for 5 min to visualize nuclei and actin filaments, respectively. The stained images were taken with an Olympus DP72 camera (Olympus Corporation, Tokyo, Japan). The adherent cell numbers and area were measured by an image measurement program (ImageJ version 1.53).

### 4.11 Cell culture on 3D scaffolds

Three‐dimensional scaffolds (∮5 mm × 2 mm h) were sterilized by treatment with non‐thermal oxygen plasma for 10 min to maintain the porous structure and immersed in the medium in the syringe. Slight syringe pumping was performed to remove the air inside the scaffolds, and then, the 3D scaffolds were inserted into the middle of a 1.5 ml microcentrifuge tube. rMSCs (1 × 10^5^) were seeded on the top of the scaffolds, and the upper part of the tube was closed with a micropipette filter to exchange the gas. Cells were incubated at 37°C in a humidified atmosphere of 5% CO_2_ overnight, and then, the 3D scaffolds were transferred into an ultralow‐attachment 96‐well plate. For cell penetration inside scaffolds, cells on scaffolds were cultured for 3 and 5 days in growth medium and then stained with phalloidin as mentioned above, and Z‐stack images were obtained using a confocal laser scanning microscope (Zeiss LSM 700, Germany). For osteogenesis, cells on scaffolds were cultured in osteogenic medium for 14 days.

### 4.12 Integrin function‐blocking assay

ISB‐P films were punched with a 6 mm diameter and sterilized by EO gas. hMSCs (2 × 10^4^ cells/100 μl) were preincubated with 10 μg/ml of function‐blocking antibodies against integrin subunits α1 (FB12), α2 (P1E6), α3 (P4C2), α4 (P4C2), α5 (P1D6), α6 (NKI‐GOH3), β1 (6S6), β2 (CD18), and β3 (25E11; Millipore, Bedford, MA) in alpha MEM separately for 15 min at 37°C in a humidified atmosphere of 5% CO_2_. The preincubated cells were seeded on ISB‐P films placed inside 96‐well plates and incubated for 1 h at 37°C in a humidified atmosphere of 5% CO_2_. Then, nuclear and actin filament staining was performed as mentioned above, and images were taken by digital microscopy (Celena S, Logos Biosystem, Inc., Anyang‐si, South Korea). The cell area was quantified using ImageJ.

### 4.13 Fibronectin adsorption assay

Rhodamine fibronectin (#FNR01, Cytoskeleton, Inc.) was reconstituted to a final concentration of 20 μg/ml (2 μg/100 μl) in PBS. Then, PCL and ISB‐P films 6 mm in diameter were individually immersed in 100 μl of working solution at 37°C. After 1 h, the fluorescence intensity of the supernatants was measured using a 535 nm excitation and 585 nm emission filter set. The adsorption amount of fibronectin was determined by subtracting the fluorescence of a group without protein (*n* = 5).

### 4.14 RNA sequencing

For RNA sequencing, 2 × 10^4^ rMSCs were seeded on PCL and ISB‐P films (16 mm in diameter) sterilized by EO gas and TCP. After 4 h, total RNA was isolated by using RibospinTM (304–150, Geneall, Seoul, Korea) according to the manufacturer's protocol. RNA sequencing was performed by DNALink, Inc. (Seoul, Korea) (*n* = 3). In brief, RNA purity was determined by assaying 1 μl of total RNA extract on a NanoDrop8000 spectrophotometer. Total RNA integrity was assessed using an Agilent Technologies 2100 Bioanalyzer with an RNA integrity number (RIN) value. mRNA sequencing libraries were produced according to the manufacturer's instructions (Illumina TruSeq Stranded mRNA Library Prep Kit). mRNA was purified and fragmented from total RNA (1 μg) using poly‐T oligo‐attached magnetic beads with two rounds of purification. Cleaved RNA fragments primed with random hexamers were reverse transcribed into first‐strand cDNA using reverse transcriptase, random primers, and dUTP in place of dTTP.^108^ Then, a single “A” base was added to these cDNA fragments, followed by ligation of the adapter. The products were purified and enriched with PCR to create the final strand‐specific cDNA library. The quality of the amplified libraries was verified by capillary electrophoresis (Bioanalyzer, Agilent). After qPCR using SYBR Green PCR Master Mix (Applied Biosystems), libraries with index tagging were combined in equimolar amounts in the pool. Cluster generation occurred in the flow cell on the cBot automated cluster generation system (Illumina). Then, the flow cell was loaded on a NovaSeq 6000 1 sequencing system (Illumina), and sequencing was performed with a 2 × 100 bp read length. The obtained raw data were analyzed using ExDEGA software (v1.6.7, E‐biogen), and significantly expressed genes with fold changes >2 and *p* values <0.1 were chosen. The 62 genes were selected and plotted in a heatmap using ExDEGA GraphicPlus (v2.0, E‐biogen, Seoul, Korea). The RNA‐seq data are available at the NCBI Gene Expression Omnibus with accession number GSE199790 (http://www.ncbi.nlm.nih.gov/geo/info/linking.html).

### 4.15 siRNA transfection

Predesigned rat and human Hspa1a‐specific siRNA duplexes (Ca. 24472‐1, 3303‐1) and nontargeting siRNA (AccuTarget™ Negative control siRNA, SN‐1003) were purchased from Bioneer (Daejeon, South Korea). Targeting (Hspa1a) siRNA and nontargeting (ctrl) siRNA were transfected individually using Lipofector‐EZ (AB‐LF‐EZ150, AptoBio, Yongin, Korea) according to the manufacturer's instructions with slight modifications. In brief, rMSCs and hMSCs were seeded at densities of 2.4 × 10^4^ cells/cm^2^ and 1.57 × 10^4^ cells/cm^2^, respectively, in growth medium. The next day, the medium was changed to alpha MEM supplemented with 10% FBS after washing with PBS. Targeting or nontargeting siRNA (1 μg/50 μl) and 2 μl/50 μl Lipofector‐EZ were prepared in alpha MEM. Diluted RNA and Lipofector‐EZ solution were mixed gently and incubated for 15 min at RT. Then, 50 μl of diluted complex was applied to each well and incubated at 37°C in a humidified atmosphere of 5% CO_2_. After 24 h, samples were collected for qRT‐PCR and cell adhesion arrays.

### 4.16 Myogenic differentiation

For static conditions, C2C12 cells were seeded (2 × 10^4^ cells/well) on films 6 mm in diameter (PCL@OP, ISB‐P sterilized by EO gas) and TCP (96‐well plates) and cultured in myogenic differentiation medium containing 5% horse serum and 1% insulin‐transferrin‐selenium (Gibco, Invitrogen, Carlsbad, CA) for 5 days or 7 days. For dynamic conditions, materials were applied to UniFlex**®** culture plates, and cells were seeded at a density of 3 × 10^5^ cells/well. Cells were cultured in differentiation conditions under dynamic tension using a Flexcell machine (Dunn Labortechnik, Asbach, Germany) with 10% strain, 0.5 Hz, and 1 h of tension stimulus each day for 4 days. At each time point, cells were washed with PBS, fixed in 4% paraformaldehyde (PFA), and permeabilized in PBS containing 0.5% (v/v) Triton X‐100 (T8787, Sigma‐Aldrich) for 10 min. After three washes in PBS, cells were incubated in PBS containing 1% (w/v) bovine serum albumin (BSA, BAH68‐1272, Equitech‐Bio, Inc., Kerrville, TX) for 1 h at RT for blocking. MHC (SC‐376157, Santa Cruz Biotechnology, Inc., Santa Cruz, CA) was diluted 1:100 in blocking solution and applied overnight at 4°C, followed by three washes with PBS. Then, the cells were incubated in the dark with a 1:100 dilution of FITC‐conjugated donkey anti‐mouse IgG antibody (715‐095‐150, Jackson ImmunoResearch Laboratories, West Grove, PA) or rhodamine (TRITC)‐conjugated antibody (715‐025‐150) for 60 min at RT. After three washes with PBS, nuclear and actin filament staining was performed as mentioned above, and the multinucleated myotubes were observed using an inverted microscope (Olympus, IX71, Tokyo, Japan) and quantified using ImageJ.

### 4.17 Osteogenic differentiation

For osteogenic differentiation, rMSCs were seeded on PCL and ISB‐P films 6 mm in diameter that had been sterilized by EO gas or on oxygen plasma‐treated 3D scaffolds and cultured in alpha MEM supplemented with 10% FBS, 1% P/S, 10 mM β‐glycerophosphate, 10 nM dexamethasone, and 50 μg/ml ascorbic acid for 7 or 14 days. The different sterilization methods were performed for matching initial cell numbers. The medium was changed every 3 days. Osteogenic gene expression (RUNX2, ALP, COL1, OCN, and BSP) was evaluated at 7 days by quantitative real‐time PCR (qRT‐PCR), and mineralization was estimated by ARS staining at day 14.

### 4.18 qRT‐PCR

Total RNA was isolated using RibospinTM (304‐150, Geneall, Seoul, Korea) according to the manufacturer's protocol, and RNA quality and quantity were analyzed in a NanoDrop 2000 spectrophotometer (ND‐2000, Thermo Fisher Scientific). One gram of total RNA (1 μg) was used for the synthesis of first‐strand cDNA using Accupower RT premix (K‐2043, Bioneer). qRT‐PCR was performed with 2X SensiMix SYBR Hi‐ROX Mastermix (QT‐605‐05, Bioline, UK) according to the manufacturer's protocol using the primers shown in Table S3. RUNX2, ALP, Col 1, BSP, OCN, and GAPDH primers were purchased from Integrated DNA Technologies, Inc. (Coralville, IA), and DNAJA1, HSP90AA1, HSPA1A, and HSPH1 primers were obtained from Bioneer. The Ct value was used to determine the efficiency of different genes with respect to glyceraldehyde 3‐phosphate dehydrogenase (GAPDH) as the endogenous reference amplified from the samples. The internal control ΔCt was calculated by (ΔCt = Ct a target gene −Ct a GAPDH), and then, the relative transcript quantities were calculated using the ΔΔCt (ΔΔCt = ΔCt a target sample −ΔCt a control sample) method. The fold change for each gene was subsequently determined from 2^−ΔΔCt^. Each measurement was performed in triplicate (*n* = 3). The primer sequences of the genes are shown in Table S3. RUNX2, ALP, Col 1, BSP, OCN, and GAPDH primers were purchased from Integrated DNA Technologies, Inc. (Coralville, IA), and DNAJA1, HSP90AA1, HSPA1A, and HSPH1 primers were obtained from Bioneer.

### 4.19 ARS staining

At 14 days, mineralization was evaluated by ARS staining. For this procedure, samples were washed with PBS, fixed in 4% PFA for 10 min, washed with deionized water (DW) and then incubated with 40 mM Alizarin red (pH 4.2; Sigma‐Aldrich) solution for 30 min at RT. Next, the samples were washed 3 times with DW and then dried at RT. The stained images were acquired using an Epson Perfection V300 photo scanner. The dye was dissolved in 10% cetylpyridinium chloride (Sigma‐Aldrich), and the absorbance was measured at 562 nm using a spectrophotometer (Varioskan LUX, Thermo Fisher Scientific, MA) (*n* = 3).

### 4.20 In vitro biocompatibility test

Human monocytes (THP1, ATCC) were differentiated into adherent macrophages by PMA (50 ng/ml, Peprotech) treatment for 24 h and seeded on PCL@OP, ISB‐P, and TCP. THP‐1 cells were lysed immediately after a 48 h polarization step, and qPCR was performed to measure inflammatory gene (TNF‐a, IL‐1b, and IL‐6) expression. Primer sequences are listed in Table S3.

### 4.21 In vivo animal studies

First, in vivo tissue responses, biocompatibility, and organ toxicity were examined in Sprague–Dawley (5‐week‐old, male) rats by subcutaneous implantation. The Animal Care and Use Committee at Dankook University, Republic of Korea (#17–011), approved the animal care and experimental protocols. The animals were adapted for 5–8 days before surgery. Each rat was housed in a cage under a controlled environment with free access to water and food provided ad libitum following guidelines. Animal surgery was performed under general anesthesia [(a mixture of ketamine (80 mg/kg) and xylazine (10 mg/kg)]. The biosafety of PCL‐PU and PCL film (8 mm diameter × 0.25 height) was tested by implantation in separated subcutaneous tissue pockets on both lateral sides of the spine, and the incision was closed by a nonabsorbable suture (4–0 Prolene, Ethicon Germany). After 2 and 4 weeks of implantation, the surrounding tissue was collected for histological analysis after immediate fixation in 10% neutral buffered formalin for 24 h at RT. And after 8 weeks of implantation, the major organs (kidney, liver, spleen, lung, and heart) were obtained to access the organ toxicity based on histological analysis. Next, a long‐term bone regeneration study was performed in a rat calvarium regular‐shape defect model established as described previously.^109^, ^110^ The calvarial skin and periosteum were exposed by a sagittal incision in the middle of the scalp under general anesthesia administered by intramuscular injection [a mixture of ketamine (80 mg/kg) and xylazine (10 mg/kg)]. A 5‐mm diameter trephine (South Korea) was used to create regular bone defects on the right and left sides of the parietal bone under cooling conditions. Then, a 3D PCL‐PU scaffold (5 mm diameter × 2 mm height) was applied to the bone defect area, the periosteum was sutured with absorbable sutures (4–0 Vicryl®, Ethicon, Germany), and the skin was folded with nonabsorbable suture material (4–0 Prolene, Ethicon, Germany). Maxillary sinus defects and long‐bone defects in rabbits were created by a high‐speed dental machine. For x‐ray analysis, iodoform in excess was dissolved in ethanol. This solution was absorbed by the porous scaffold and then removed. Before implantation, the scaffold it was pressed until it was half its uncompressed height, as confirmed by x‐ray analysis. The defect width was the same as the half‐height of the scaffold, and the length was the same as the scaffold width. After 1 h of surgery, x‐ray imaging was used to confirm that the cavity was filled with scaffold. Micro‐CT (μ‐CT) imaging and histological analysis were conducted 8 weeks after the operation. The animals were euthanized by CO_2_ inhalation. The defect sites were dissected, fixed in 10% neutral buffered formalin (NBF), and subsequently scanned by a μCT instrument (Skyscan 1176, Skyscan, Aartselaar, Belgium). After the scans, bone visualization was performed with 3D iso‐surface renderings, and new BV, BS, and BSD values were determined.

### 4.22 Histological and immunohistochemical analysis

All the samples were dehydrated in an ethanol series, embedded in paraffin, and sectioned at 5‐μm thickness.^111^ Especially the tissue samples obtained from calvarial defects were completely decalcified in RapidCal™ solution (BBC Chemical Co.) before dehydration. For histology analysis, the tissue slides were stained with hematoxylin and eosin (H&E) and Masson's trichrome (MT) and visualized by light microscopy after hydration. Inflammation scores were further determined by pathologist using high‐magnified H&E stained images.^112^ For IF examination, the sections were incubated with primary antibodies targeting iNOS (ab15323, Abcam), TNF‐α (52B83, Santa Cruz), CD206 (6A598, Santa Cruz), and Arg‐1 (ab203490, Abcam) to detect proinflammatory and anti‐inflammatory markers. Secondary antibodies conjugated with rhodamine (Alexa Fluor 594) and FITC (Alexa Fluor 488) and DAPI were applied for counterstaining. The IF‐stained sections were observed using a fluorescence microscope (IX71, Olympus, Japan), and quantitative analysis was performed using ImageJ software.

## AUTHOR CONTRIBUTIONS

**Suk‐Min Hong:** Conceptualization (equal); data curation (equal); methodology (equal); writing – original draft (equal). **Ji‐Young Yoon:** Conceptualization (equal); data curation (equal); formal analysis (equal); investigation (equal); methodology (equal); writing – original draft (equal). **Jae‐Ryung Cha:** Conceptualization (equal); data curation (equal); formal analysis (equal); writing – original draft (equal). **Junyong Ahn:** Data curation (equal); formal analysis (equal); investigation (equal). **Nandin Mandakhbayar:** Data curation (equal); formal analysis (equal); funding acquisition (equal); methodology (equal). **Jeong Hui Park:** Data curation (equal); investigation (equal). **Junseop Im:** Formal analysis (equal); funding acquisition (equal); investigation (equal). **Gangshi Jin:** Data curation (supporting); investigation (supporting); resources (supporting). **Moon‐Young Kim:** Data curation (equal); funding acquisition (equal). **Hae‐Hyoung Lee:** Conceptualization (equal); data curation (equal); funding acquisition (equal). **Jung‐Hwan Lee:** Conceptualization (equal); supervision (equal); writing – review and editing (equal); funding acquisition (equal). **Hae‐Won Kim:** Conceptualization (equal); supervision (equal); writing – review and editing (equal); funding acquisition (equal).

## CONFLICT OF INTEREST

The authors declare no conflict of interest.

## Supporting information


**Appendix S1** Supporting information

**Video S1** Stability of the suture interface between tissue and ISB‐P

**Video S2** Shape memory of a thread form at 37.5°C

## Data Availability

The data that support the findings of this study are available from the corresponding author upon reasonable request.
