# Supplementary material for: Hyperelastic, shape‐memorable, and ultra‐cell‐adhesive degradable polycaprolactone‐polyurethane copolymer for tissue regeneration
Source: Bioeng Transl Med. 2022 May 5;7(3):e10332. doi: 10.1002/btm2.10332 (PMC9472029; doi:10.1002/btm2.10332)
Supplement: Supplementary file 1 — Appendix S1 Supporting information [file BTM2-7-e10332-s002.docx]

**Hyperelastic, shape-memorable and ultra-cell-adhesive degradable polycaprolactone-polyurethane copolymer for tissue regeneration**

Suk-Min Hong^1,2,3,#^, Ji-Young Yoon^1,2,5,11,#^, Jae-Ryung Cha^1,2,3,#^, Junyong Ahn^1,2,4,5^, Nandin Mandakhbayar^1,2,5^, Jeong Hui Park^1,5^, Junseop Im^7^, Gangshi Jin^1,2,5^, Moon-Young Kim^1,5,8^, Jonathan C. Knowles^2,5,9,10^, Hae-Hyoung Lee^1,2,4,5^, Jung-Hwan Lee ^1,2,4,5,6*^, Hae-Won Kim ^1,2,4,5,6*^

^1^Institute of Tissue Regeneration Engineering (ITREN), Dankook University, Cheonan, Chungcheognam-do, 31116, Republic of Korea

^2^Department of Nanobiomedical Science and BK21 PLUS NBM Global Research Center for Regenerative Medicine, Dankook University, Cheonan, Chungcheognam-do, 31116, Republic of Korea

^3^Department of Chemistry, College of Science and Technology, Dankook University, Cheonan, Chungcheognam-do, 31116, Republic of Korea

^4^Department of Biomaterials Science, College of Dentistry, Dankook University, Cheonan, Chungcheognam-do, 31116, Republic of Korea

^5^UCL Eastman-Korea Dental Medicine Innovation Centre, Dankook University, Cheonan, Chungcheognam-do, 31116, Republic of Korea

^6^Cell & Matter Institute, Dankook University, Cheonan, Chungcheongnam-do, 31116, South Korea.

^7^Samyang Corporation, 730 Daeduck-daero, Yuseong-gu, Daejeon, 34055, South Korea

^8^Department of Oral and Maxillofacial Surgery, College of Dentistry, Dankook University, 119 Dandae-ro, Cheonan, Chungcheongnam-do 31116, Republic of Korea

^9^Division of Biomaterials and Tissue Engineering, Eastman Dental Institute, Royal Free Hospital, Rowland Hill Street, London, NW3 2PF, UK

^10^The Discoveries Centre for Regenerative and Precision Medicine, Eastman Dental Institute, University College London, London, UK

^11^TCell & Matter Corporation, Cheonan, Chungcheongnam-do, 31116, South Korea.

----------------------

^#^These authors contributed equally to this work.

*Corresponding authors:

- Jung-Hwan Lee (ducious@dankook.ac.kr)

- Hae-Won Kim ([kimhw@dku.edu](mailto:kimhw@dku.edu))

- Address: Institute of Tissue Regeneration Engineering (ITREN), Dankook University, Republic of Korea

**Supplementary information**

**Table S1. Summary of the composition of PCL-diol-HDI-based polyurethane depending on isosorbide derivative**.

| **Polyurethanes** | **HDI (g)** | **PCL diol**  **(M_w_ 2000) (g)** | **Isosorbide (g)** | **Ethoxylated isosorbide** | **Propoxylated isosorbide** | **Yield (%)** |
| --- | --- | --- | --- | --- | --- | --- |
| mole ratio / weight (g) | | | | | | |
| ISB-P | 10 / (10) | 5 / (59.457) | 0 / (0) | 0 / (0) | 5 / (12.854) | 96.9 |
| ISB-E | 10 / (10) | 5 / (59.457) | 0 / (0) | 5 / (10.499) | 0 / (0) | 97.5 |
| ISB-2′ | 10 / (10) | 5 / (59.457) | 5 / (4.344) | 0 / (0) | 0 / (0) | 91.1 |
| ISB-free | 10 / (10) | 10 / (118.913) | 0 / (0) | 0 / (0) | 0 / (0) | 98.5 |

**Table S2. Molecular weights (M_n_, M_w_) and polydispersity indices of PCL and PCL-PUs determined by gel permeation chromatography. The second value of PCL in parenthesis was provided by manufacturer (Sigma, Cat. Num. 440744).**

|  | **M_n_ (g/mol)** | **M_w_ (g/mol)** | **PDI** |
| --- | --- | --- | --- |
| ISB-E | 123,786 | 142,586 | 1.152 |
| ISB-P | 139,744 | 159,354 | 1.14 |
| ISB-2′ | 50,986 | 103,336 | 2.027 |
| ISB-free | 70,586 | 89,534 | 1.268 |
| PCL | 85,658  (80,000) | 224,120  (<160,000) | - |

**Table S3. Primer sequence.**

| **Genes** | **Forward primers (5′-3′)** | **Reverse primers (5′-3′)** |
| --- | --- | --- |
| RUNX2 (rat) | GACCGACACAGCCATATA | TCCCTAACCTGAAACCAAAG |
| ALP (rat) | CTCTGCCGTTGTTTCTCTAT | AGGTGCTTTGGGAATCTG |
| Col 1 (rat) | CTGGTACATCAGCCCAAAC | GAACCTTCGCTTCCATACTC |
| BSP (rat) | GGACACTTACCGAGCTTATG | CCTTCACTGGTGGTAATAAT |
| OCN (rat) | GCTTCAGCTTTGGCTACT | CGTTCCTCATCTGGACTTTAT |
| DNAJA1 (rat) | GATTAAGGAGGGCGGAGCAG | ACTGAGAGCTGATGCACGAC |
| HSP90AA1 (rat) | TTCCAACTCCTCAGACGCTC | GGGTTCGGTCTTGCTTGTTG |
| HSPA1A (rat) | CCTGAGCAAGGAGGAGATCG | CATGTTGAAGGCATAGGACTCG |
| HSPH1 (rat) | GTTGAGCTGCCTGTTGAAGC | CGGCGTTCTTAGCATCGTTC |
| GAPDH (rat) | CAAGGATACTGAGAGCAAGAG | ATGGAATTGTGAGGGAGATG |
| HSPA1A (human) | GCTGACCAAGATGAAGGAG | GCTCAAACTCGTCCTTCTC |
| Integrin α5 (human) | GGACTGTGGAGAAGACAACATC | GTGAGGTTCAGGGCATTCTT |
| TNF-α (human) | TGCTTGTTCCTCAGCCTCTT | CAGAGGGCTGATTAGAGAGAGGT |
| IL1-β (human) | CCAGGGACAGGATATGGAGCA | TTCAACACGCAGGACAGGTACAG |
| IL-6 (human) | ACAGCCACTCACCTCTTCAG | CCATCTTTTTCAGCCATCTTT |
| GAPDH (human) | GGA GTC CAC TGG CGT CTT CAC | GCT GAT GAT CTT GAG GCT GTT GTC |

**sVideo 1. Stability of the suture interface between tissue and ISB-P**

**sVideo 2. Shape memory of a thread form at 37.5°C.**


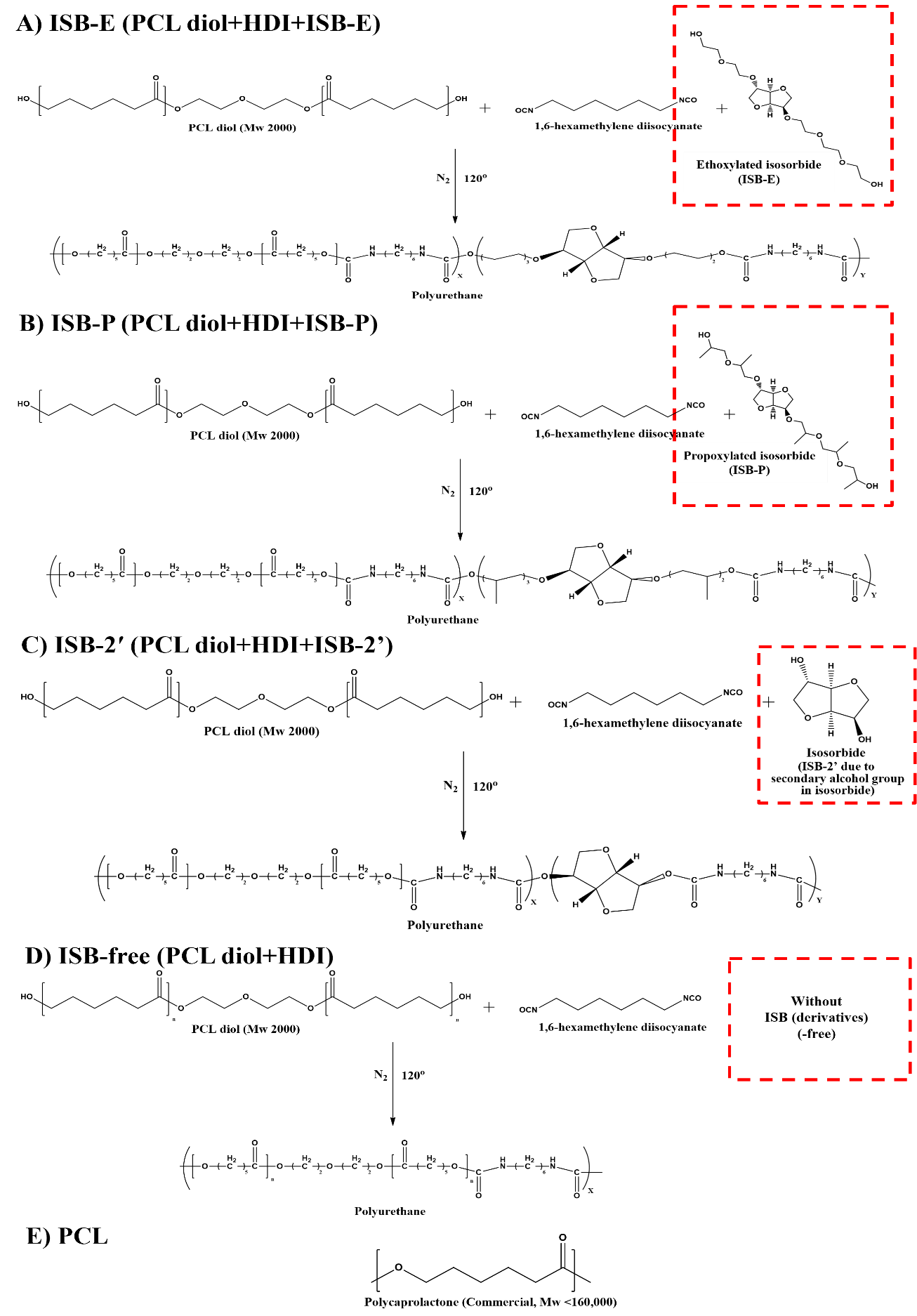


**sFig. 1** **Schematic structure of PCL-PU derivatives and PCL.** A) ISB-E from PCL diol, hexamethylene diisocyanate1,6-hexamethylene diisocyanate (HDI), and ethoxylated isosorbide (E), B) ISB-P from PCL diol, HDI, and propoxylated isosorbide (P), C) ISB-2′ from PCL diol, HDI, and bare isosorbide (with secondary alcohol group; 2’), D) ISB-free from PCL diol and HDI without isosorbide (derivatives) and E) commercially available high-molecular weight PCL (Mn 80,000 and Mw < 160,000).


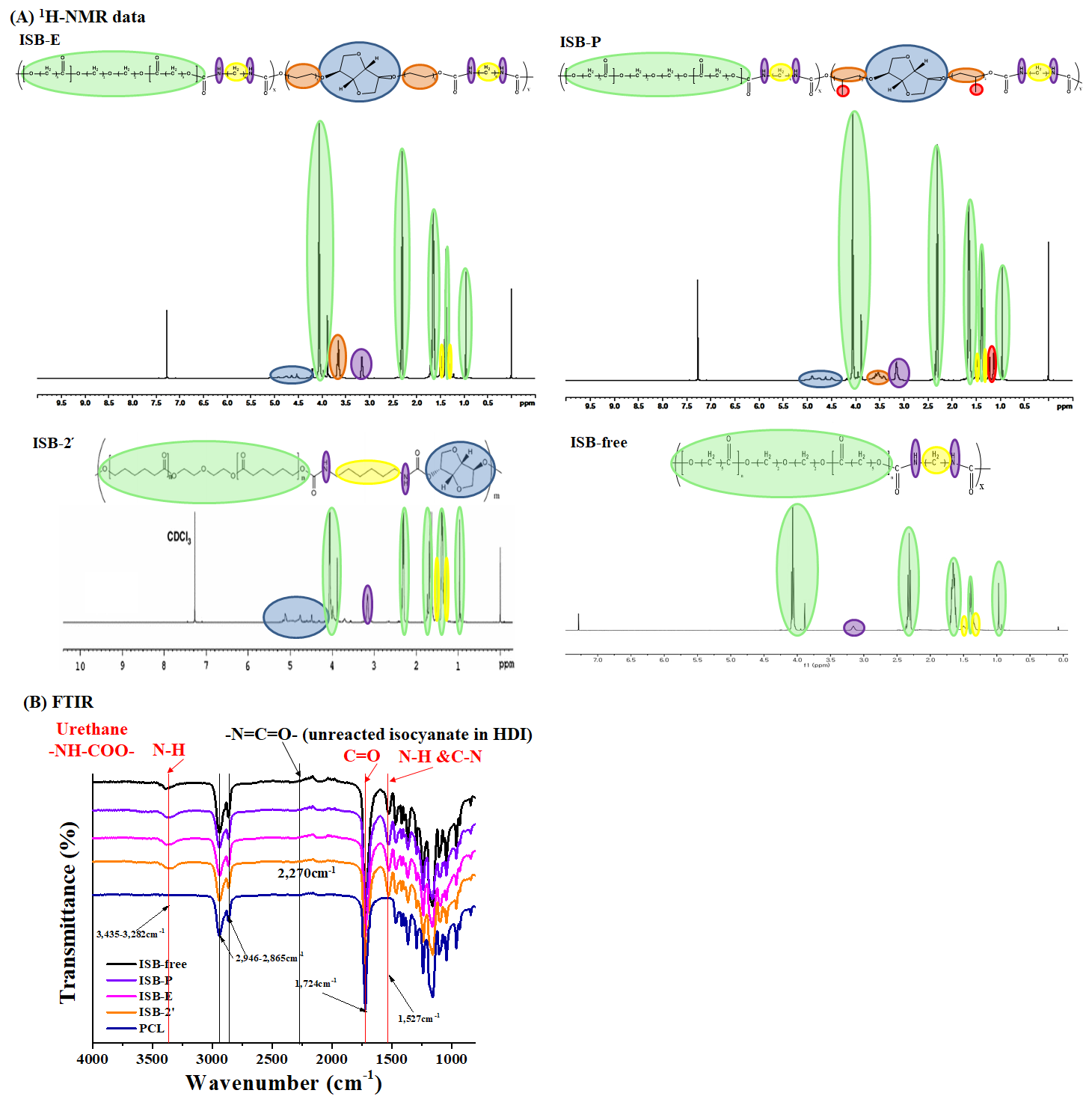


**sFig. 2. Confirmation of synthesized PCL-PUs.** A) ^1^H-NMR spectra of ISB-E, ISB-P, ISB-2’ and ISB-free B) and FT-IR spectra of PU derivatives and PCL. ^1^H-NMR spectra of ISB-2’ was re-used from our previously study ^1^. Urethane (-NH-COO-) peaks (red line) were successfully detected in all PCL-PU while unreacted isocyanate peak from HDI was not detected.


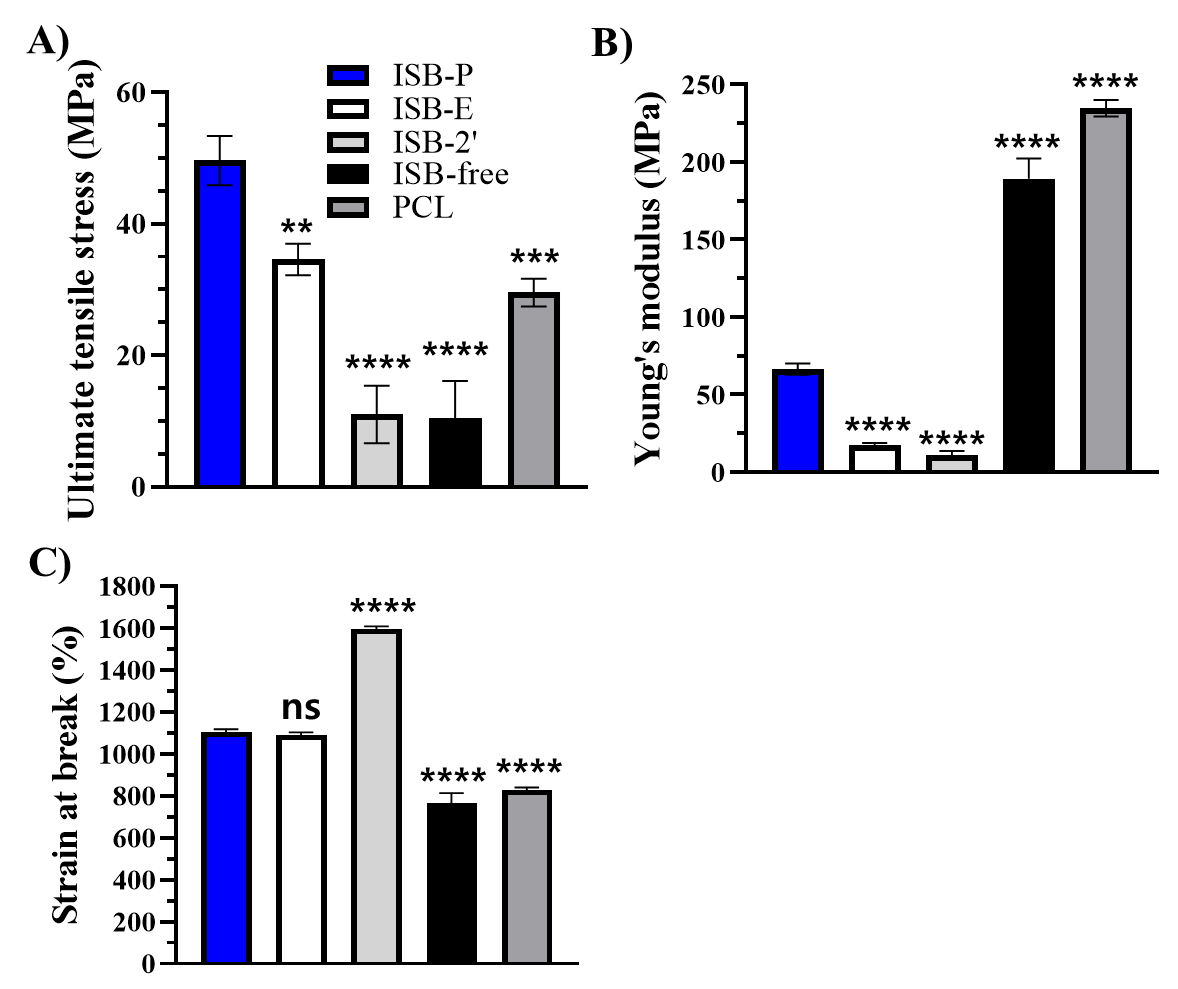


**sFig. 3. Mechanical properties calculated based on the stress-strain curve.** A) Stress-strain curve measured by a universal testing machine, B) ultimate tensile stress, C) Young`s modulus, and D) strain at break (%) (n=5, ** <0.01, ***<0.001, ****<0.0001 compared to ISB-P, One-way ANOVA with Dunnett`s tests, ns (no significant difference)).


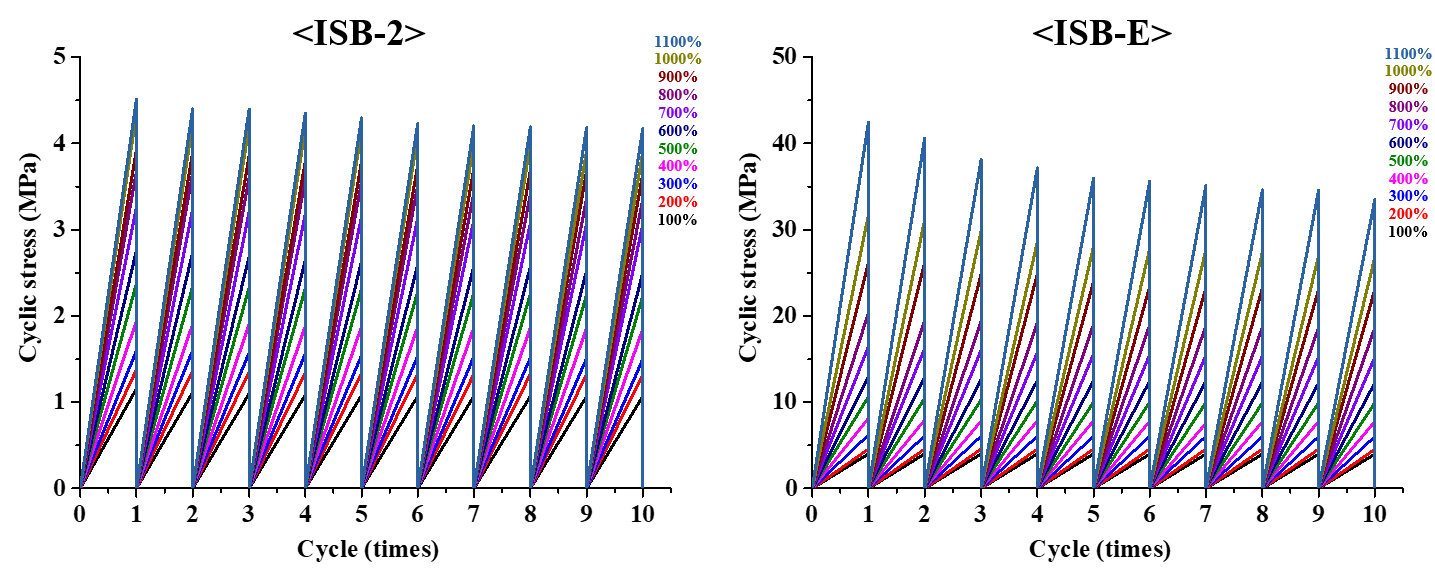


**sFig. 4. Cyclic tensile test result using ISB-2 and ISB-E up to 10 cycles.** ISB-free was excluded from this cyclic tensile study due to low elasticity.


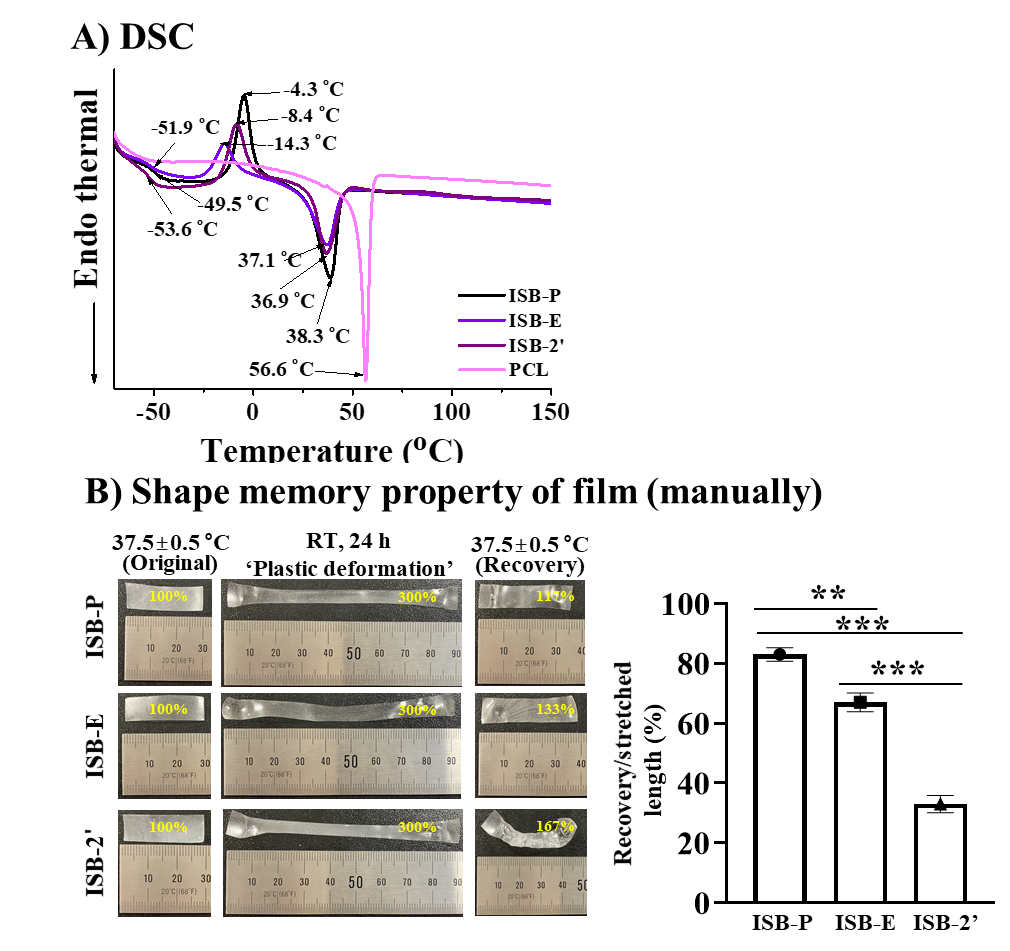


**sFig. 5. Shape memory property of PCL-PUs.** A) Tm values investigated by DSC for determining theoretical memory transfer temperature for inducing shape-memory change. B) Bulk shape memory property under tension was performed on film, revealing the most recovery property in ISB-P than others (n=3, **P<0.01, ***P<0.001 by one-way ANOVA with Dunnett`s test).


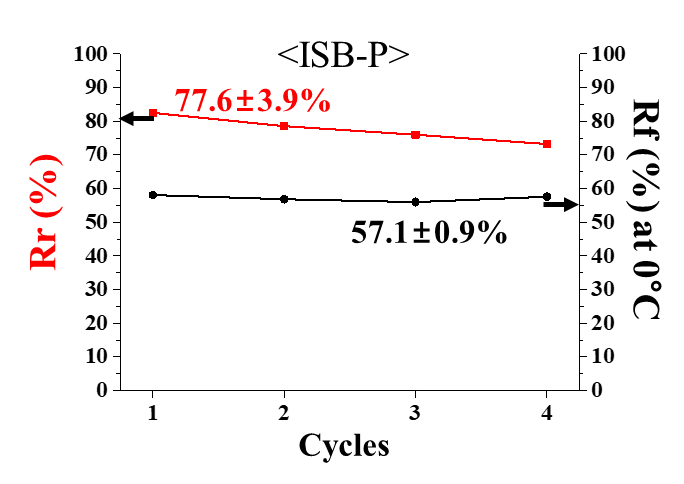


**sFig. 6 Shape recovery ratio (R_r_) and shape fixing ratio (R_f_) of ISB-P, measured by a thermomechanical cyclic experiment.**


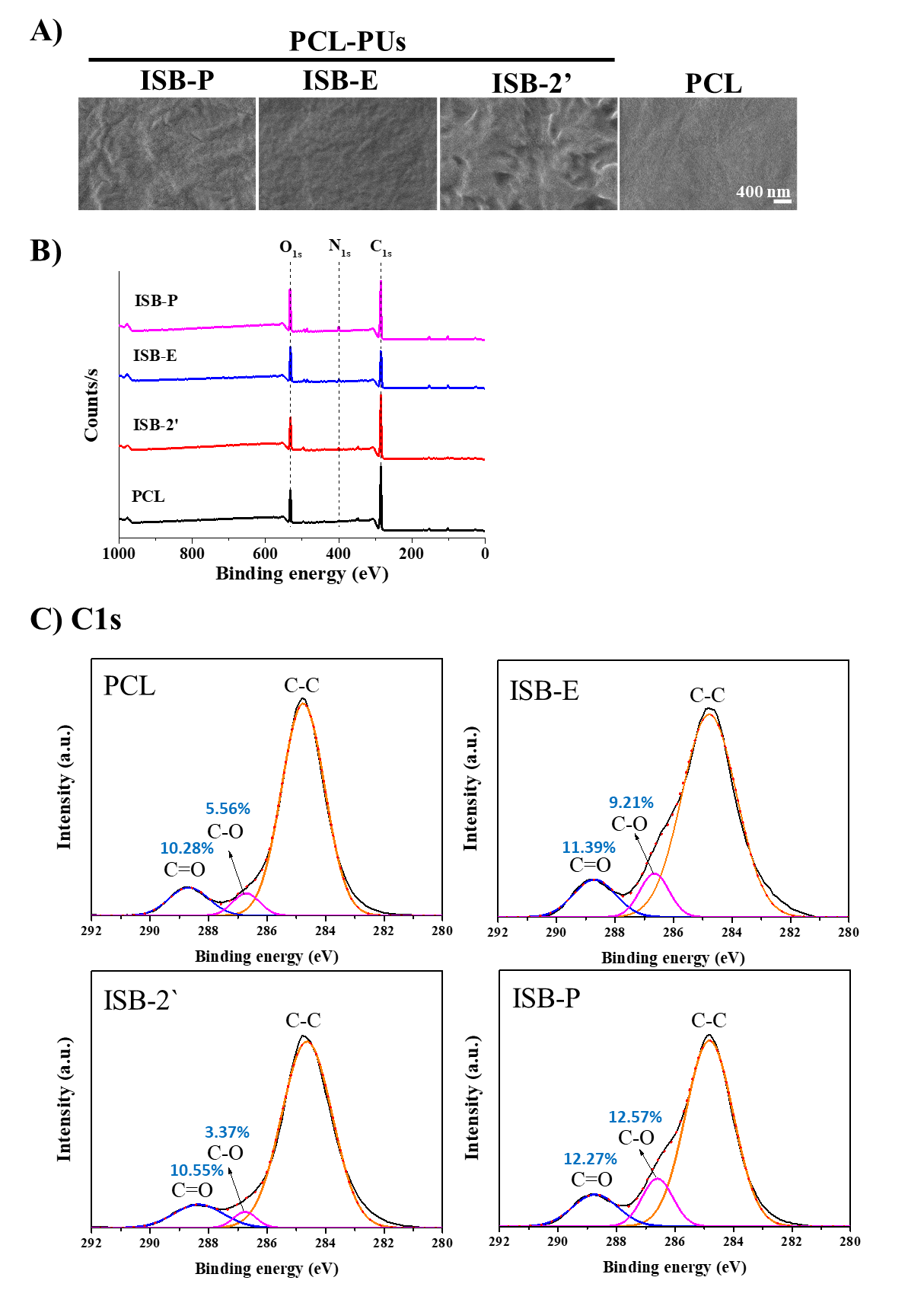


**sFig. 7. Surface characterization.** (A) Visualization by SEM and (B-C) chemical bonding analysis by XPS. After the survey scan, the C1s peak was analyzed in detail.


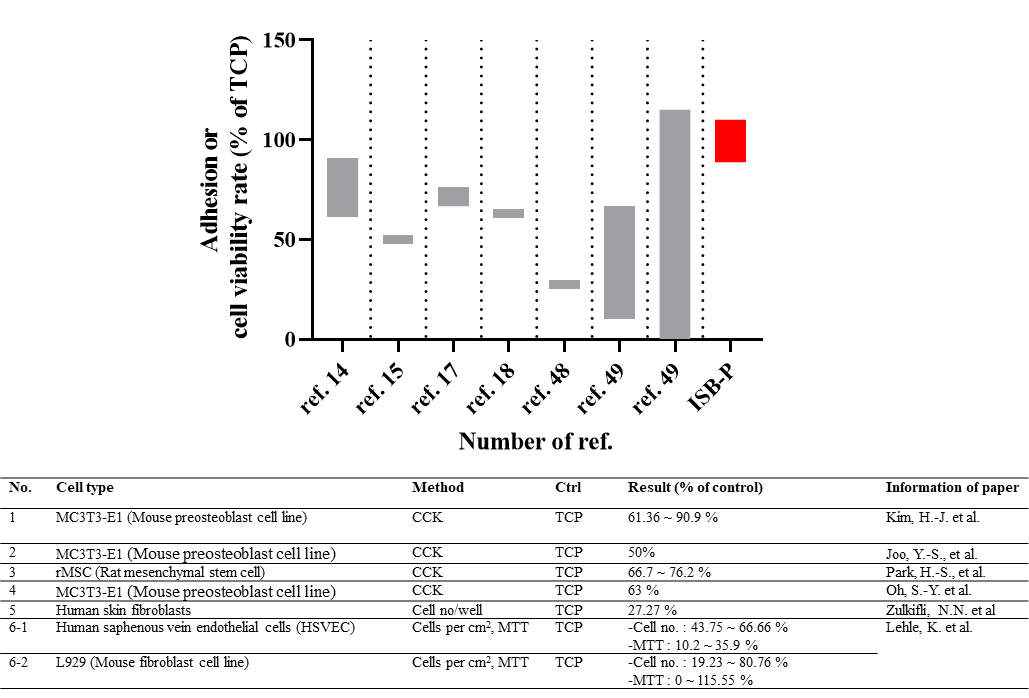


**sFig. 8. Cell-adhesive properties of PU compared to those of TCP according to other literatures ^1-6^ versus those of ISB-P, confirmed by the results of CCK, MTT or cell adhesion area analysis.** ISB-P showed higher stability and stronger hyperadhesion than TCP.


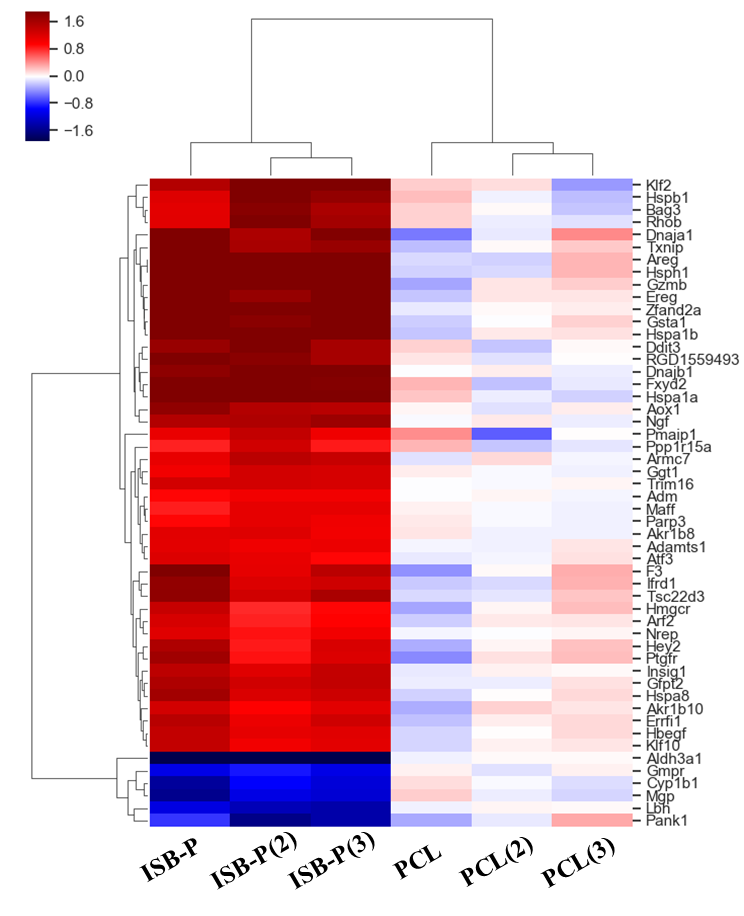


**sFig. 9. Heat map of genes with upregulated and downregulated expression from triplicate RNA sequence analysis (n=3).** Sixty-two significantly different genes with over twofold changes from normalized values over 16 were visualized.


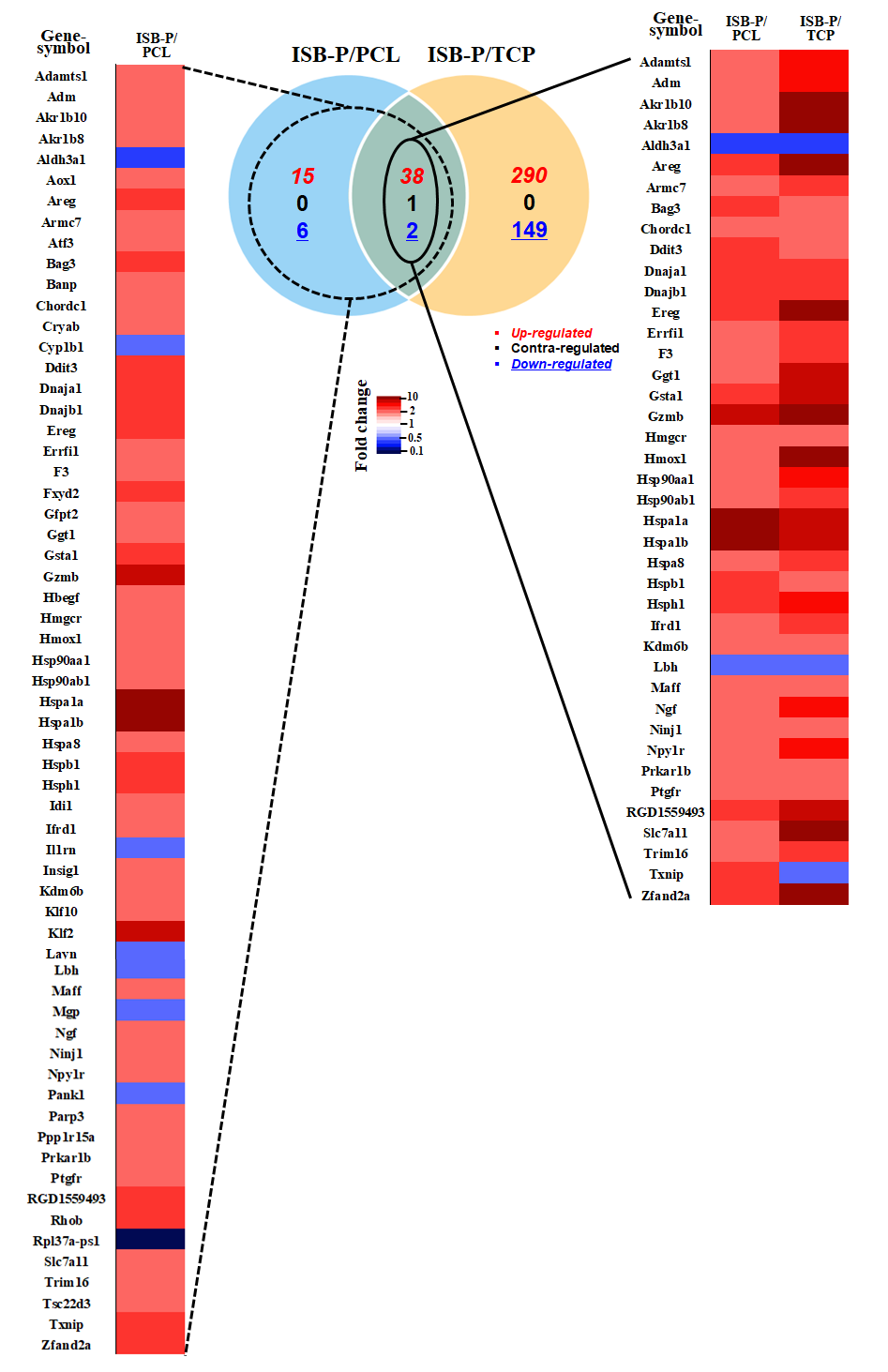


**sFig. 10. Venn diagram showing the overlap of differentially expressed genes (DEGs) detected by RNA sequencing among the 3 groups.** Red, black, and blue indicate genes with up, contra- or downregulated expression between groups, respectively. The list of DEGs with colored fold changes is shown in detail.


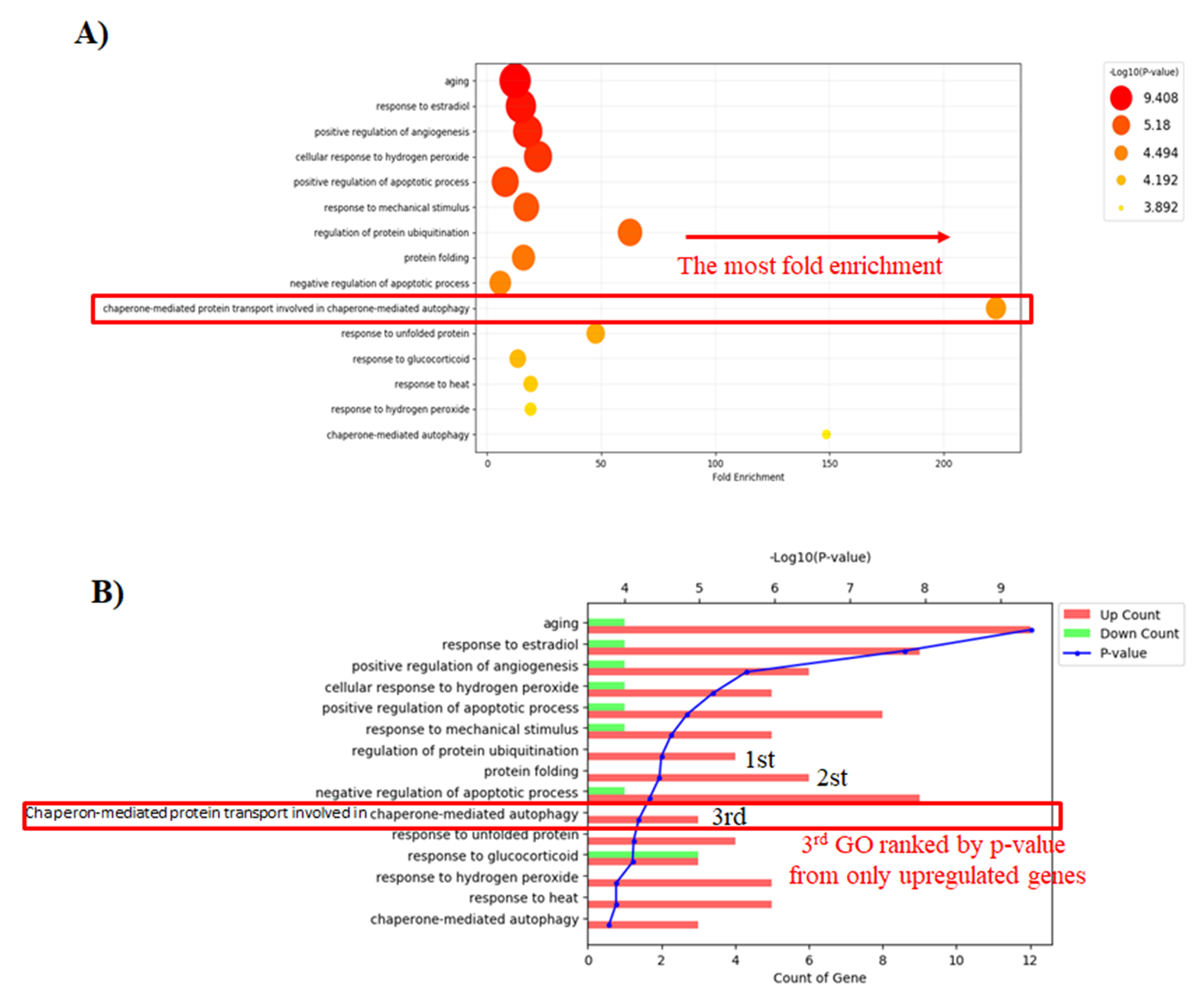


**sFig. 11. Top enriched Gene Ontology terms by DAVID analysis of 62 genes with up- or downregulated expression in ISB-P with respect to PCL.** The most fold enrichment GO term was ‘Chaperone-mediated protein transport involved in chaperone-mediated autophagy’, indicating the possible involvement of chaperone-mediated genes in ISB-P’s cell-adhesiveness.


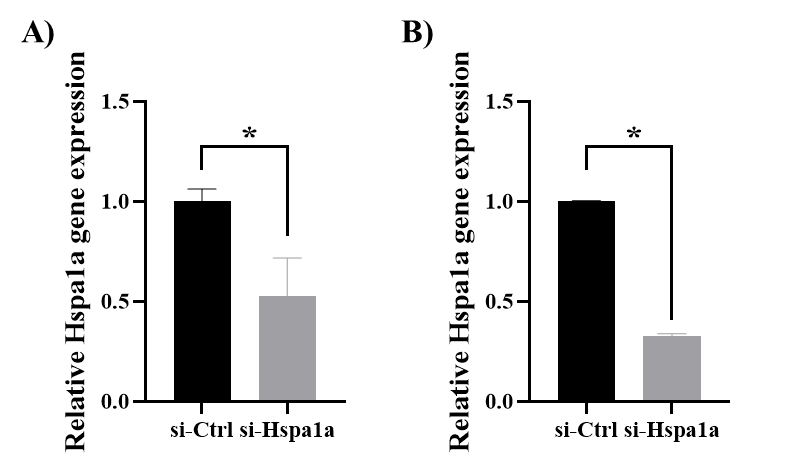


**sFig. 12. Hspa1a gene knockdown in rMSCs (A) and hMSCs (B) by si-Hspa1a confirmed by qRT-PCR.** Hspa1a gene knockdown by si-Hspa1a was confirmed by qRT-PCR. *P<0.05, by t-test.


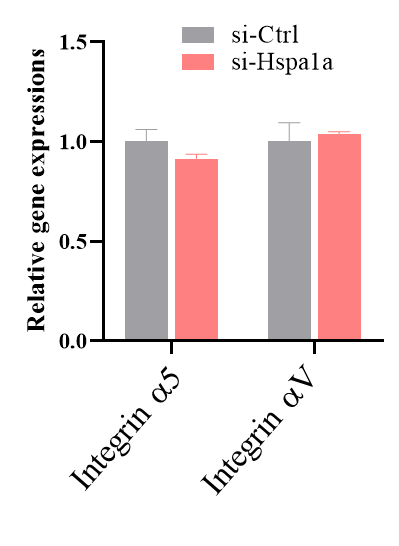


**sFig. 13. *α5* and *αν* integrin gene expression in si-Hspa1a-treated hMSCs.** It showed no change of transcription level of integrin *α5* and *αν* compared to the si-Ctrl-treated group (n=3, P>0.05).

**sFig. 14. The adsorption amount of fibronectin was revealed using rhodamine-conjugated fibronectin.** After 1 h of incubation with 20 µg/ml fibronectin solution on specimens in 96-well plates, the adsorption amount of fibronectin was calculated by the fluorescence intensity difference between the initial and supernatant solutions (n=5, P>0.05).


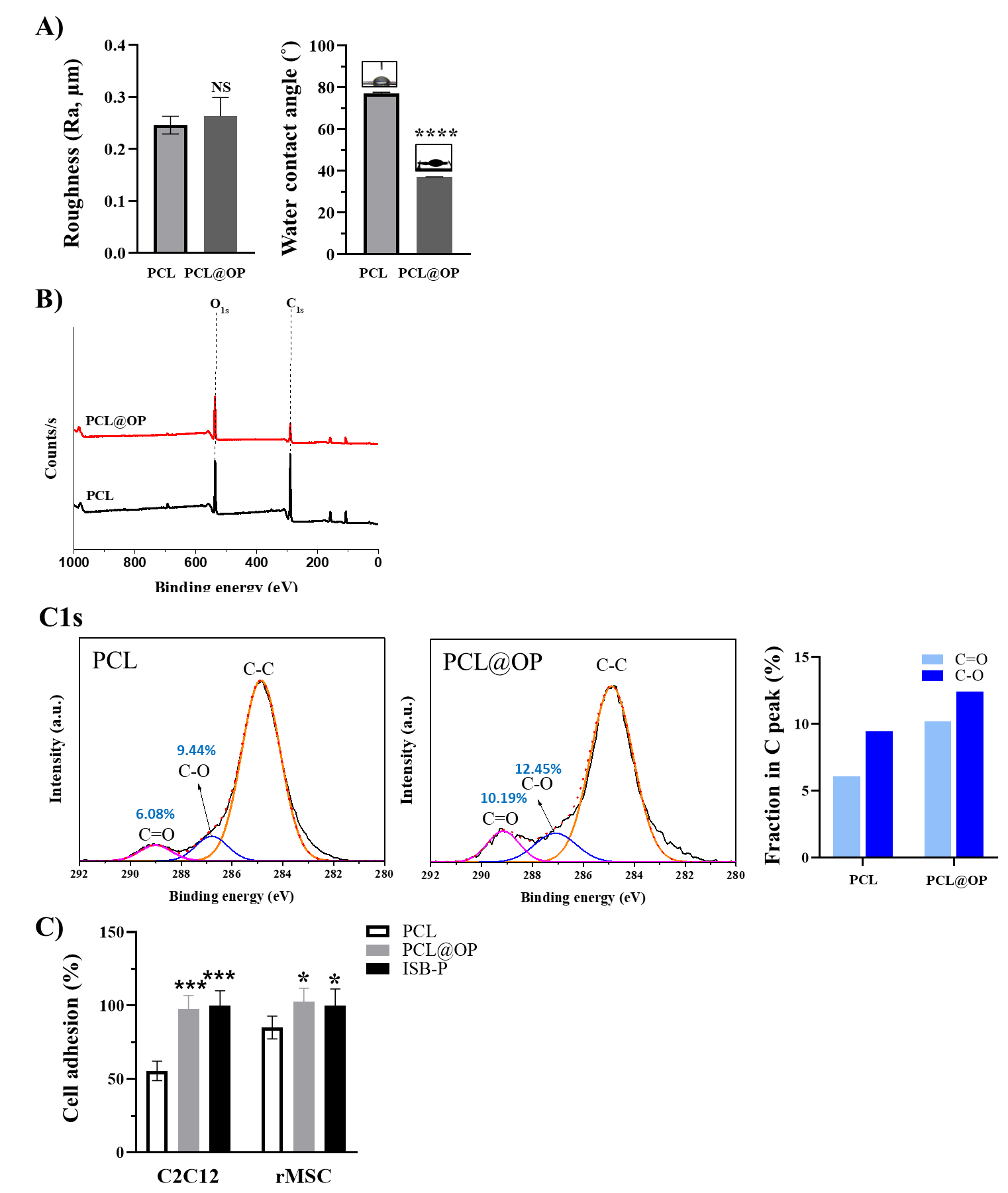


**sFig. 15. Comparison of surface characteristics and cell adhesion before and after oxygen-plasma treatment.** (A-B) Surface characteristics. A) Roughness (n=3), water contact angle (n=3), ****P<0.0001 by unpaired t test. B) Survey spectra and the high-resolution XPS spectra of C1s peak. (C) Cell adhesion (%) comparison among PCL, PCL@OP, and ISB-P. C2C12 and rMSC were seeded respectively on substrate and adherent cell numbers after 24 h were normalized to that of bare ISB-P (n=5). *P<0.05, ***P<0.001 compared to bare PCL, by 2-way ANOVA with Dunnett`multiple comparisons test. No statistical difference between PCL@OP and ISB-P (P>0.05).


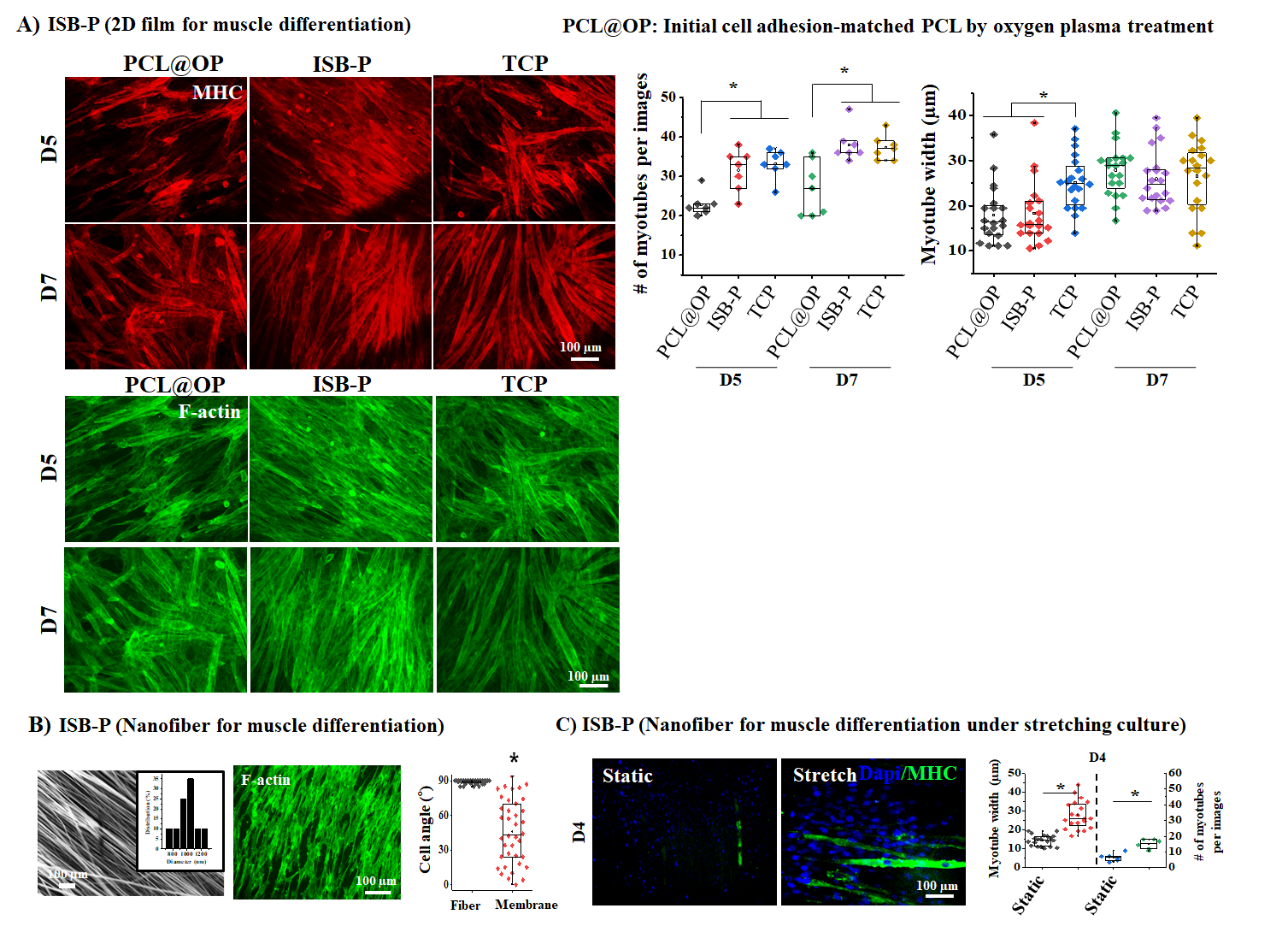


**sFig. 16. Myogenic differentiation of C2C12 on PCL-PU (ISB-P) for tissue engineering.** A) Representative images immunostained for MHC (red) and actin (green) after myogenic induction of C2C12 for 5 and 7 days on initial adhesion-matched ‘oxygen plasma treated PCL (PCL@OP)’, ISB-P, and TCP under 2D static conditions. Quantification of the number and width of MHC-stained myotubes, indicating the differentiation-conductive properties of ISB-P (n=7 or 20). B) C2C12 adhesion at 24 h was visualized by F-actin (green) staining, and the cell alignment was calculated along with the nanofiber direction. SEM image of unidirectionally electrospun ISB-P nanofibers 700–1300 nm in diameter was given. The distribution of diameter is shown in the insert image. C) Dynamic culture of nanofiber ISB-P for myogenic differentiation. C2C12 cells were seeded on aligned nanofibers and dynamically cultured for 4 days (4 s stretching for 10% and 6 s resting per cycle, 1 h per day). MHC (green) and nuclei (blue) were visualized and quantified (n=6 or 20). Nanofiber ISB-P under stretching conditions is more myogenic than that under static conditions. *P<0.05 by t-test or one-way ANOVA with Dunnett`s tests.


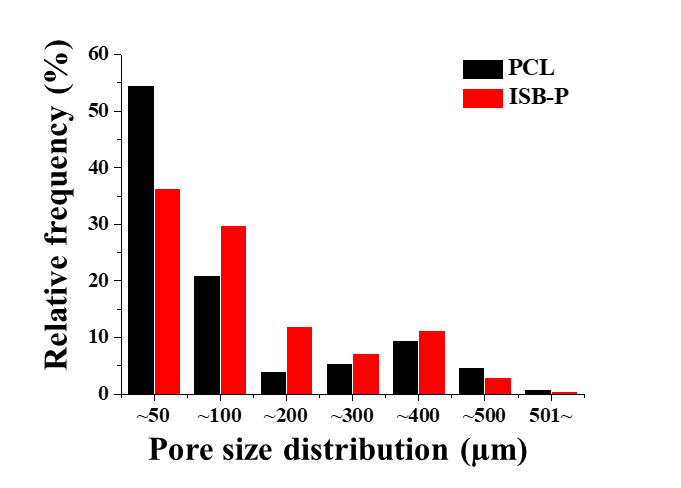


**sFig. 17. Three-dimensional scaffold pore size distribution (n=400).**


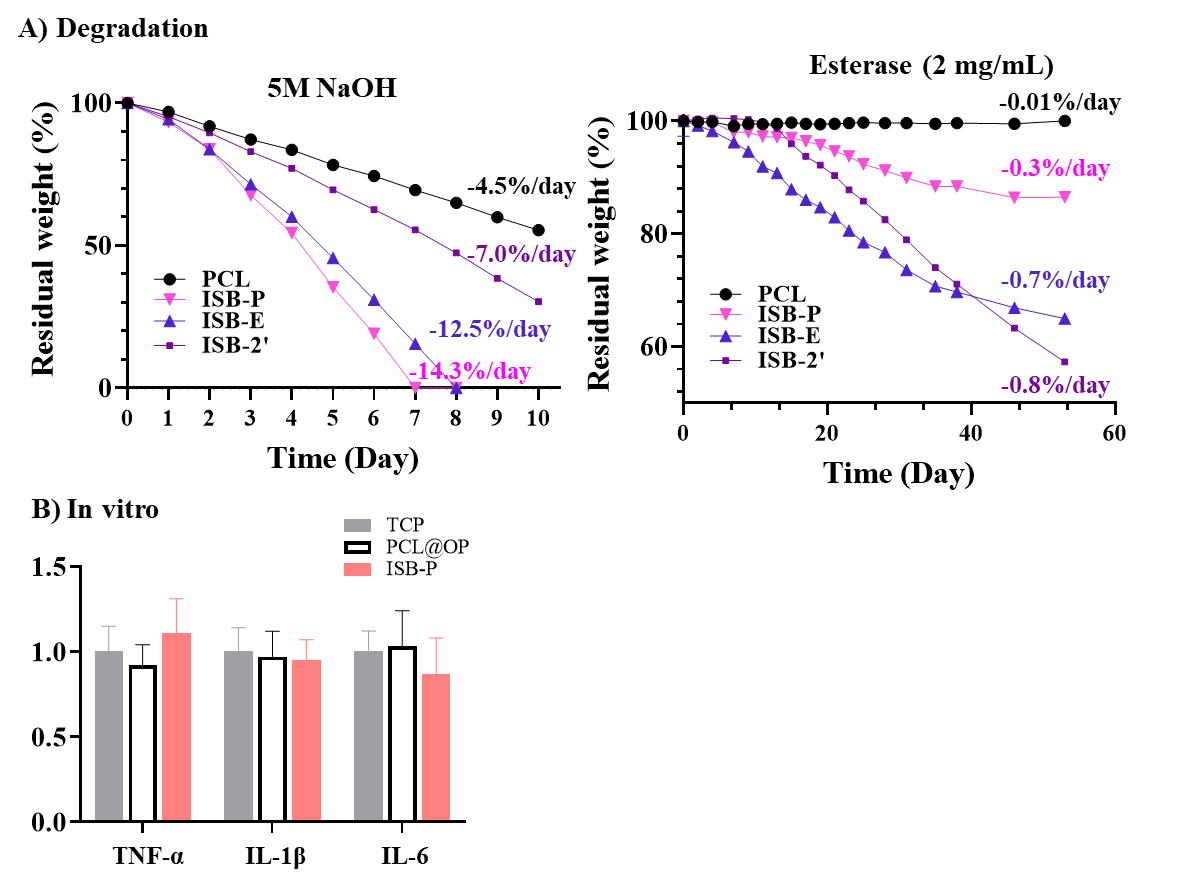


**sFig. 18. Degradation of PCL-PU and in vitro immune response study.** A) Chemical and enzymatic degradation studies using NaOH (5M) and esterase (2 mg/ml) were performed at 37°C with 120 rpm for 10 and 53 days (n=3) respectively. B) THP1 cells, a human macrophage cell line, were used for investigating the initial immune response. After M0 polarization by PMA (50 ng/ml phorbol 12-myristate 13-acetate), activated THP1 cells were seeded on the substrate, and inflammatory gene (TNF-a, IL-1a, and IL-6) expression was investigated by qPCR analysis after 24 hr of seeding. ISB-P and PCL@OP showed no difference in inflammatory gene expression (n=5,).


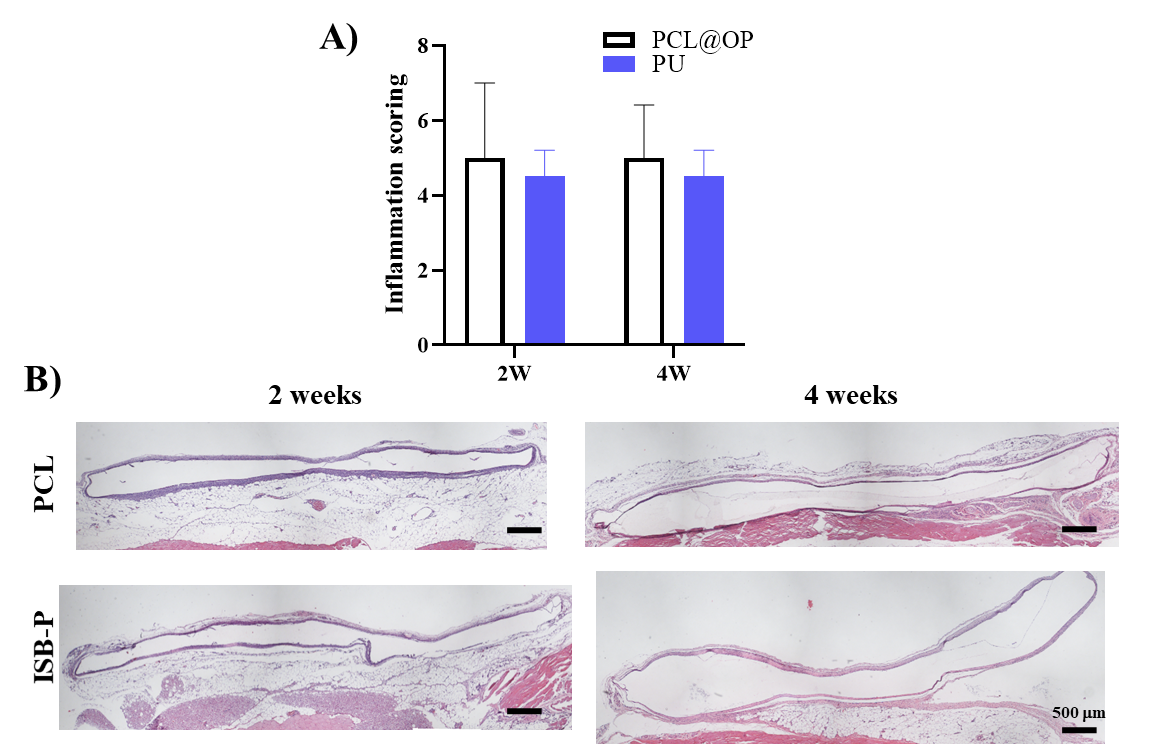


**sFig. 19. Subcutaneous biocompatibility test.** A) Inflammation scoring calculated by histological analysis. B) H&E stained full-image of the implanted materials and surrounding tissues at 2 and 4 weeks.


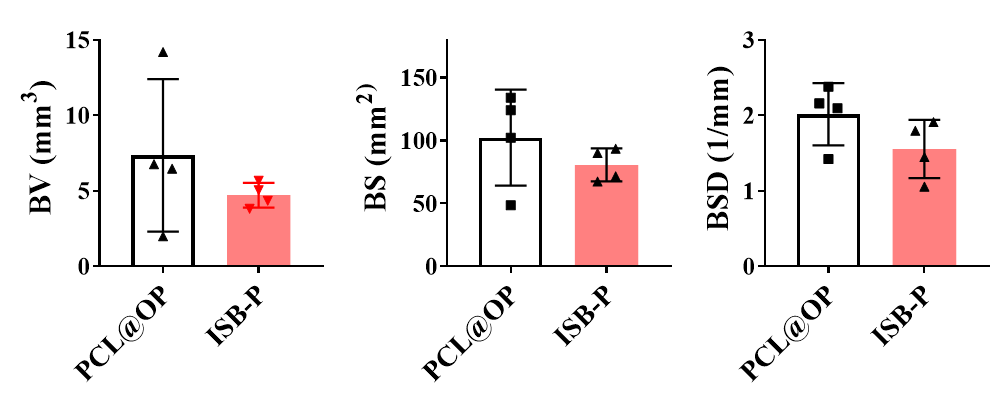


**sFig. 20. Regenerated bone quantified by µCT analysis from PCL@OP- and ISB-P-implanted calvarial defects at 8 weeks.**

**References**

1. Park H-s, Gong M-S, Knowles JC. Catalyst-free synthesis of high elongation degradable polyurethanes containing varying ratios of isosorbide and polycaprolactone: physical properties and biocompatibility. *Journal of Materials Science: Materials in Medicine*. 2013/02/01 2013;24(2):281-294. doi:10.1007/s10856-012-4814-0

2. Kim H-J, Kang M-S, Knowles JC, Gong M-S. Synthesis of highly elastic biocompatible polyurethanes based on bio-based isosorbide and poly (tetramethylene glycol) and their properties. *Journal of biomaterials applications*. 2014;29(3):454-464.

3. Joo Y-S, Cha J-R, Gong M-S. Biodegradable shape-memory polymers using polycaprolactone and isosorbide based polyurethane blends. *Materials Science and Engineering: C*. 2018/10/01/ 2018;91:426-435. doi:https://doi.org/10.1016/j.msec.2018.05.063

4. Oh S-Y, Kang M-S, Knowles JC, Gong M-S. Synthesis of bio-based thermoplastic polyurethane elastomers containing isosorbide and polycarbonate diol and their biocompatible properties. *Journal of biomaterials applications*. 2015;30(3):327-337.

5. bt Zulkifli NN, Amin KAM. Palm kernel oil-based polyester polyurethane composites incorporated with multi-walled carbon nanotubes for biomedical application. *Bioresources and Bioprocessing*. 2016;3(1):1-9.

6. Lehle K, Stock M, Schmid T, Schopka S, Straub RH, Schmid C. Cell‐type specific evaluation of biocompatibility of commercially available polyurethanes. *Journal of Biomedical Materials Research Part B: Applied Biomaterials*. 2009;90(1):312-318.
